# Challenges for Safe Electrolytes Applied in Lithium-Ion Cells—A Review

**DOI:** 10.3390/ma14226783

**Published:** 2021-11-10

**Authors:** Marita Pigłowska, Beata Kurc, Maciej Galiński, Paweł Fuć, Michalina Kamińska, Natalia Szymlet, Paweł Daszkiewicz

**Affiliations:** 1 Faculty of Chemical Technology, Institute of Chemistry and Electrochemistry, Poznan University of Technology, Berdychowo 4, PL-60965 Poznan, Poland; marita.piglowska@student.put.poznan.pl (M.P.); maciej.galinski@put.poznan.pl (M.G.); 2 Faculty of Civil Engineering and Transport, Institute of Combustion Engines and Powertrains, Poznan University of Technology, Berdychowo 4, PL-60965 Poznan, Poland; pawel.fuc@put.poznan.pl (P.F.); michalina.kaminska@put.poznan.pl (M.K.); natalia.szymlet@put.poznan.pl (N.S.); pawel.daszkiewicz@put.poznan.pl (P.D.)

**Keywords:** non-flammable electrolyte, safety LIBs, polymer electrolytes, SEI, hydrogen

## Abstract

The aspect of safety in electronic devices has turned out to be a huge challenge for the world of science. Thus far, satisfactory power and energy densities, efficiency, and cell capacities have been achieved. Unfortunately, the explosiveness and thermal runaway of the cells prevents them from being used in demanding applications such as electric cars at higher temperatures. The main aim of this review is to highlight different electrolytes used in lithium-ion cells as well as the flammability aspect. In the paper, the authors present liquid inorganic electrolytes, composite polymer–ceramic electrolytes, ionic liquids (IL), polymeric ionic liquids, polymer electrolytes (solvent-free polymer electrolytes (SPEs), gel polymer electrolytes (GPEs), and composite polymer electrolytes (CPEs)), and different flame retardants used to prevent the thermal runaway and combustion of lithium-ion batteries (LIBs). Additionally, various flame tests used for electrolytes in LIBs have been adopted. Aside from a detailed description of the electrolytes consumed in LIBs. Last section in this work discusses hydrogen as a source of fuel cell operation and its practical application as a global trend that supports green chemistry.

## 1. Introduction

The key to maintain a safe and high-performance lithium-ion battery inheres in the identification of a suitable electrolyte [1]. Electrolytes used in LIB have to meet a variety of expectations: low vapor pressure, low melting points, and high boiling points (allowing a large operating temperature range). Favorable transport properties (fast transport of lithium ions between anode and cathode) and chemical and electrochemical stability (preserving the electrolyte during the charge–discharge process) also play an important role [2]. The following properties are also important: ionic conductivity (high lithium-ion cation rate to achieve high power), salt solubility/crystalline solvates (the use of low-temperature cells in particular), and solid electrolyte interphase (SEI) formation (preventing further electrolyte–electrode reactions). The aluminum corrosion used as the current collector is also significant: a specified electrolyte has to passivate the electrolyte–Al interface in order to prevent corrosive pitting of the collector [3,4]. In the case of selecting the electrolyte, appropriate properties such as the dielectric constant, viscosity, conductivity, density, and volatility are very important. The solvent that is used to obtain the electrolyte is most often a mixture of organic liquids (which, unfortunately, are flammable) [5].

The greatest challenge for scientists is reducing the flammability of lithium-ion cells to the greatest possible extent. A truly important parameter is the thermal capacity defined as the heat that can be absorbed by an object. It is well known that, for effective heat dissipation, the designs should allow for both the cell and the battery back levels. It heavily depends on the surface area, size, and geometry of the battery. Even if the heat dissipation is well designed, the small hotspots within the battery could lead to an explosion [6], which is why every little detail plays a significant role when it comes to thermal capacity and thus safety of the cell. The thermal runaway process consists of three main stages: the onset of overheating (stage 1), the heat accumulation/gas release process (stage 2), and the combustion and explosion (stage 3). In order to solve the problems during stage 1, reliable anode materials, multifunctional liquid electrolytes, and separators are used for overcharging protection. In order to overcome the problems during stage 2, reliable cathode materials, thermally switchable current collectors, thermal shutdown separators, separators with a high thermal stability, and battery packs with cooling functions are applied. In order to fix the problems during stage 3, nonflammable liquid electrolytes or flame retardants are used [7]. There exists a classification of nonflammable electrolytes [8]:

- Organic electrolytes with nonflammable parts such as co-solvents or various additives for instant organic phosphorous compounds; they exhibit a high conductivity and good electrochemical performance;
- Polymeric solid electrolytes consisting of polymer complexes with lithium salts for instant LiX/poly (ethylene oxide) (PEO); they exhibit a low conductivity at lower temperatures;
- Polymeric gel electrolytes with polymer complexes, which are swollen in organic solvents for instant LiX/alkylcarbonate/PEO with a nonflammable component; they exhibit high ionic conductivity;
- Ionic liquids consisting of ionic liquids (ILs) dissolving lithium salt for instant LiX/IL, IL: 1-ethyl3methylimidazolium fluorosulfonyl amide (EMIFSA); they exhibit a high ionic conductivity and low-rate capability;
- Inorganic solid electrolytes constructed from lithium ion-containing oxides, sulfides, glass, and ceramics (superionic conductor Li_10_GeP_2_S_12_ crystal); they have a high ionic conductivity and a high mobility of lithium ions.

With the increasing battery capacity, ensuring the safety of the LIB becomes very demanding. Additives that are typically used in nonaqueous electrolytes (liquid organic, ionic liquids, polymer, inorganic solid, inorganic liquid, liquid organic + polymer, ionic liquid + polymer + liquid organic, polymer + inorganic solid, ionic liquid + liquid organic [9]) can be classified according to their different functions [10]:

- Function-improving additives;
- Additives for safety improvement (e.g., anisole compounds, halogenobenzene compounds, alkylbenzene compounds for overcharge prevention) and phosphate and phosphazene compounds for non-flammability;
- Miscellaneous additives (e.g., corrosion inhibition);
- Additives for anodes (e.g., carboxylic acid anhydrides, oxalates);
- Additives for cathodes (e.g., sulfur-containing or aromatic compounds).

**Figure 1:**
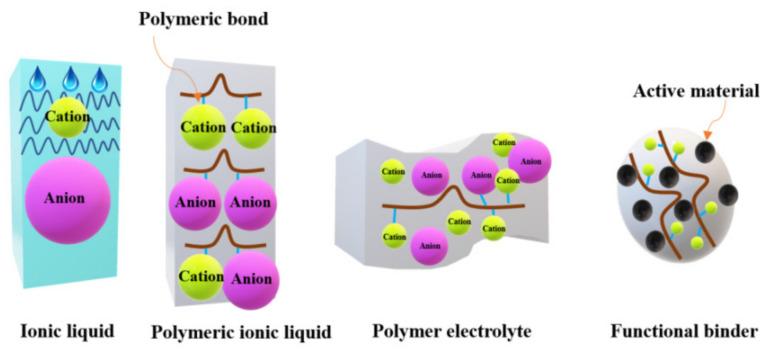
A schematic representation of ionic liquids, polymeric ionic liquids, polymer electrolytes, and functional binders.

Figure 1 shows the schematic structures of different electrolytes for LIBs: the ionic liquid, the polymeric ionic liquid, and the polymer electrolyte [11].

These electrolytes gradually replace classic electrolytes such as lithium hexafluorophosphate (LiPF_6_). They increase thermal stability, ensure lower explosiveness, increase safety, and quantify ionic conductivity. Additionally, oxygen reacts with carbonate electrolytes, which generates substantial heat [12]. Lithium-based storage devices of both high-power and high-energy densities are necessary for electric devices, particularly for electric vehicles/hybrid electric vehicles as well as portable electric devices [13]. Therefore, safety issues play an important role in ensuring their controllable use.

## 2. Results and Discussion

### 2.1. Safety Issues and Conventional Security of LIBs

In order for the cell to work safely, it is necessary to pay attention to the following aspects during its operation and design: thermal instability, dendritic lithium, overcharging, and gas evolution (especially at high temperatures). The following factors influence the thermal hazard: physical (during a vehicle collision), electrical (external short-circuit during contact with water or following overcharging), thermal as well as manufacturing defects, and aging processes. The thermal factor is caused by overheating that leads to the melting of the separator, decomposition of the electrodes and electrolyte and, consequently, the thermal runaway. Electrical and mechanical factors also lead to thermal escape. This is possible because the change in the internal energy occurs through heat, work, and radiation from the thermodynamic point of view. Manufacturing defects may include, for example, poor quality of the separator, insufficient purity of materials, or inappropriate arrangement of the cell components, which leads to a cell malfunction.

In order to increase the energy density, thinner separators are built; however, this increases the possibility of short-circuiting the battery. In order to prevent breakdowns, the use of flame retardant (FR) separators is recommended. They must meet requirements in terms of porosity; thermal, mechanical, and chemical stability; wettability; and separation capabilities. The FR separator usually consists of composite materials, e.g., those containing the addition of aluminum compounds, bromine, or cellulose [14,15,16,17,18].

Overcharging consists of three stages (based on the example of a cobalt or graphite electrode in a classic lithium salt electrolyte). In the first stage, charging takes place starting from the voltage of 2.8–4.2 V. Intercalation into the graphite structure and deintercalation from the cobalt salt structure take place along with a change of the free intercalation/deintercalation enthalpy without changing the crystal structure. In the second stage, the voltage increases beyond the assumed one, which causes excessive deintercalation of the lithium ions from the cathode until their complete removal. This will change the crystal structure and then the cobalt deposit on the anode, which causes irreversible capacity. In the third stage, the electrolyte and the organic solvent start to decompose, causing the emission of gases that increase the pressure in the cell. Eventually, the temperature rises drastically, leading to overcharging and eventually an explosion [19].

**Figure 2:**
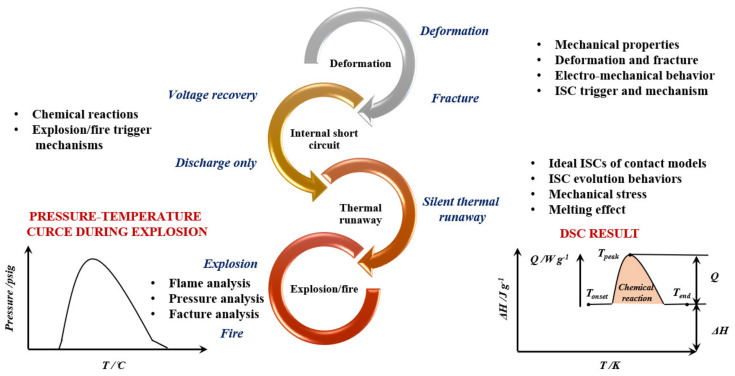
Typical milestone events of four phases: deformation, internal short circuit (ISC), thermal runaway, and explosion/fire, based on.

Mechanical wear of a cell is one of the most common causes of cell failure that can lead to an explosion. In addition to mechanical causes, electrochemical and thermal factors also play an important role (Figure 2) [20]. Excess current levels can damage the cell; hence, it is extremely important to protect it against overvoltage and all transients. An important aspect when choosing electrolytes are the costs and the possibility of further commercialization. Some of the important safeguards are vents, fuses, and switches. There is a spike at the top of the cell that pierces the diaphragm when internal pressure increases. This allows gases to escape through the vents and prevents the cell from breaking. These openings may be replaced with devices with a positive temperature coefficient. Thermal fuses play an important role in keeping the cell safe. They work on the principle of self-destruction while protecting the device, thanks to which they open the circuits permanently. They are referred to as impulse discharges that may blow fuses prematurely.

In addition to the above-mentioned safeguards, there are also magnetic switches and bimetallic thermostats used to protect power supplies. They are used to observe the load current and temperature. Thermistors are divided into those with a positive temperature coefficient and those with a negative one. The advantage is that the incoming current can be controlled. Thermostats are used to terminate charging or discharging, and they operate at a constant temperature. Due to the application of electrolytes in the organic solvent, LIBs require external protection systems against overcharging or over-discharge [21]. Other solutions to safety issues are redox shuttle additives, flame retardants, or positive temperature coefficient devices [22].

Film formation on the surface of electrodes is a very common phenomenon in electrochemical systems. Most of the metallic electrodes in both aqueous and non-aqueous solutions are covered in a certain potential range with a top layer of film, which affects their electrochemical behavior. When a certain thickness is reached, the formed coating becomes an electronic insulator; therefore, any conductivity may result from the migration of ions through the corrosive layer under the influence of an electric field. The created film can have both anionic and cationic conductivity [23].

When considering the phenomenon of SEI formation on the electrode surface and the role of lithium-ion cells, focus should first be on the metallic lithium, because there are some similarities in surface phenomena on active metallic electrodes and carbon materials. Initially, the surface of the lithium is covered with a two-layer film formed from an inner oxide part and an outer part containing hydroxides and carbonates, as a result of the inevitable reactions of the metal with the elements’ weather conditions during the production process. After introducing the metal into polar aprotic solutions, there is an exchange reaction during which part of the primary layer dissolves or reacts with the components of the solution. The electrolyte also penetrates the film layer and reacts with the metal. Such behavior leads to the formation of a complex and non-uniform layers surface, with a multilayer structure and side structures with mosaic structure, on a submicroscopic and even nanometric scale. Lithium salts form a thin layer thanks to conductive Li ions, with which these ions can migrate through the formed coating under the influence of the electric field. However, an intense metal dissolution process can lead to damage of the SEI layer.

This results in a patchy distribution of the supplied load and the heterogeneity of the electrochemical processes taking place. As a result of damage to the SEI layer, the exposed active metal surfaces react violently with the electrolyte to form dendrites [23], which can perforate the separator, leading to electrode short circuit. This behavior largely eliminates the use of lithium metal in lithium-ion cells as an electrode material.

Extensive studies of Li–C electrodes in various electrolytes using spectroscopic techniques have shown that their surface chemistry is generally similar to that analyzed on lithium and precious metals that have been polarized using the same electrolyte solutions [24,25,26]. SEI formed on the surface of the graphite electrode during lithium insertion also has a multi-layer structure. The lithium-ion intercalation in the graphite electrode is usually done by electroplating, which means that the surface forms of the layers are formed in a highly selective process. We observe that the reaction involves first the more reactive ones, which are reduced at higher potentials. This was confirmed by the research carried out when analyzing the graphite surface at different potentials [27].

It is commonly believed that the SEI layer on the graphite anode is formed as a result of the decomposition of electrolyte components during the first stage of charging [28]. This coating inhibits the further degradation of the solvents and allows for intercalation of Li ions between the graphite layers. Additionally, it plays a beneficial role in improving the safety and cyclicality of the cell, although it is also the main cause of capacity decline by consuming a significant amount of charge during its formation. The size of the irreversible capacity depends on the composition of the electrolyte and electrode material, mainly on the type of coal used. Because the reactions take place at the surface of the particles of the material, materials with a smaller specific surface area usually show lower irreversible capacity. Numerous actions have been taken in order to find a good solvent system that will form SEI with minimal charge consumption. Various techniques have been used for this purpose to thoroughly understand the composition of SEI, its morphology, stability, creation mechanism, and its impact on the cell’s efficiency [29,30,31,32,33,34].

The demarcation of the boundary between the end of the SEI and the beginning of the electrolyte is almost impossible, which makes it difficult to represent the actual SEI image inside the cell. In that case, reference can be made to the SEI layer models discussed in the literature [35,36,37,38,39].

Layer composition and thickness do not remain constant during cyclic operation and during storage [40], because there are many different ways in which they can be changed, e.g., by partial dissolution in electrolyte [41]. SEI thickness may also change during the cyclic operation as it is believed to be thicker at low potentials (carbon intercalation state) and thinner at higher potentials (deintercalated state) [42]. The potential at which the SEI formation process begins is not constant. Various values can be found in the literature, such as 2 V, 1.7 V, and 1 V [43], although the most commonly accepted potential is 0.8 V vs. Li/Li^+^ [42]. As previously mentioned, this process takes place during the first charge of the cell, although it may be continued during the next few cycles. It should be remembered that this parameter depends on many factors, such as the type and composition of the electrolyte, the type of additives used for the electrolyte, and the speed of the charge/discharge process [43,44]. The desired effect is the complete creation of the SEI before starting the lithium intercalation process (>0.3 V vs. Li/Li^+^ [45]). This is more difficult to achieve in the case of disordered coals, because for some of them, the insertion process starts at approximately 1.5 V vs. Li/Li^+^, compared to structured carbons with an insertion potential of approximately 0.25 V [46]. Thus, the stabilization of the graphite electrode surface is achieved if the reduction of electrolyte components takes place before the intercalation of lithium, leading to the formation of a passivation layer. In contrast to lithium electrodes, the surface and volume of which largely change during galvanostatic charge/discharge processes, volumetric changes in the case of graphite during insertion/deinsertion of lithium ions are small. As a result, isolated compact groups of compounds that adhere well to the graphite surface are sufficient for effective electrode passivation, and thus lead to highly reversible cycling [47].

In conclusion, an ideal SEI should have the maximum Li^+^ conductivity. The process of creating an SEI should be completed before Li^+^ ion intercalation begins. A perfect SEI should have a uniform morphology as well as composition. A good SEI should be a compact and well-adhered layer. It should be flexible [48] and resilient [49] to accommodate non-uniform electrochemical behavior and degassing of active material.

It is worth mentioning that, for example, ionic liquids (used as electrolytes) have a relatively poor ability to form an effective SEI layer at the graphite anode, which results in a decrease in capacity during cyclic operation [50]. An interesting approach to stabilizing an intercalated graphite electrode is the application of small amounts of highly active additives, which may help to form SEI [51,52], providing protection against further electrolyte reduction on the graphite surface [53]. Examples of the additives used are carbonate ethylene (EC), EC chloride (Cl-EC), ethylene (IV) sulfate (ES), ethylene carbonate vinyl (VEC), and vinylene carbonate (VC) [53,54,55,56]. The mentioned compounds or their mixtures show the ability to form SEI on the surface of the electrodes with a stabilizing effect; among them, VC turned out to be exceptionally effective [53], and therefore it is the most commonly used additive of electrolytes in lithium-ion cells [57,58,59,60,61].

The aging of the battery, particularly the carbon anodes, is a factor that causes the cell to self-discharge and increases the impedance, thus shortening the battery life. The formation of SEI during electrolyte decomposition leads to a decrease in power. The increase in the SEI is intensified by higher temperatures and cyclic work. Parallel to the film formation, lithium corrosion occurs, resulting in a decrease in power and capacity as well as self-discharge through the loss of mobile lithium. In addition, the decomposition of the binder is intensified by high temperatures, which ultimately results in the loss of lithium and the loss of mechanical stability. This is prevented by the selection of an appropriate binder. The high temperatures also enhance the reduction of the available surface area by continuously forming SEI, which increases the impedance of the entire cell. This is prevented by ensuring the formation of a stable SEI layer by the use of appropriate additives [62].

An important element of the cell is a separator that allows certain ions to pass and prevents electrical contact between the electrodes. It provides security. The most commonly used are microporous polyolefin membranes [63]. Their disadvantage is high thermal shrinkage at higher temperatures, which can lead to internal short circuits. This then results in an increase in the temperature of the cell, and then its explosion [64]. Over the years, much attention has been paid to improving the performance of membranes, especially for active electrode nanomaterials, such as Sn alloys or Si nanoparticles. These nanomaterials undergo huge changes in volume in the lithiation and delithation processes (by about 200–300%), which significantly affects the formation of mechanical stress on the membranes. Fatigue of the materials causes them to crack after a certain number of cycles. This happens as a result of overly large changes in the volume associated with the subsequent insertion and deinsertion processes of lithium ions. Due to the lack of protection of the electrode materials against the electrolyte, both gain access to each other, which causes unfavorable pulverization or graining processes. It also results in a loss of performance. After some time, when the grains become too small, the link is no longer able to work.

Polyethylene microporous membranes were once used for car batteries used to start the engine (lead–acid cells). Over time, they began to be used in lithium batteries. The membranes used for this are mainly polyethylene microporous membranes, polyamide non-woven fabrics, gel sheets, and hydrophilically reinforced polypropylene non-woven fabrics. Since the commercialization of lithium-ion cells began, microporous membrane separators have found practical application, which also contributed to the improvement of the separators market. Their main application has been in mobile devices (smartphones, laptops). Polymers such as polyamide, polypropylene, and polyethylene, as well as paper and cellulose are also popular in the form of foil or non-woven fabrics.

In commercial cells (not counting new polymer solid cells), non-aqueous electrolytes are used, so the separator must meet requirements such as solvent resistance, thinness, and current breaking properties within a specified temperature range. When using solid, polymer, and gel electrolytes, no separators are used, which reduces production costs. Thus, the most commonly used solution is the PE microporous membrane due to meeting all the requirements, which is why it has gained the greatest application in LIBs.

### 2.2. Flammable Liquid Inorganic Electrolytes

One of the very important elements of the cell is the electrolyte. Its role allows ions to move in a certain direction: between the cathode and the anode. The materials used to obtain the electrolyte are those characterized by high conductivity, thanks to which the movement of lithium ions is continuous.

Liquid inorganic electrolytes are mostly used in lithium-ion cells. They are well established but fail to meet many criteria for commercial battery electrolytes. The most common are salts, as shown in Table 1. Commonly used in commercial batteries is LiPF_6_, in which the presence of hydrofluoric acid (HF) in the salt has a huge impact on the cell performance and is one of the concerns related to the application of this salt [65,66,67,68].

During the discharging/charging process of lithium-ion cells, there is a movement of charges. This takes place in the liquid phase of the electrolyte (when we are talking about the movement of ions) and in the solid phase (electric charge). Additionally, one can observe the movement of lithium within two phases: electrolyte and electrodes. All analyzed processes have a significant impact on the efficiency of the lithium-ion cell. The deterioration of the efficiency (capacity of the cell) is influenced by the reduction in the concentration of lithium ions in the electrolyte (which is observed when we have much higher than the ion transport speed).

During the operation of lithium-ion cells (i.e., discharging/charging processes) we deal with an electrochemical reaction. Wear or accumulation of atoms or lithium ions can be noticed. This takes place at the interface between solid active materials and the electrolyte. Transport takes place on the principle of diffusion and migration (this happens within the electrode and electrolyte material).

Numerous literature reports confirm that the amount of electrolyte significantly affects the energy density and capacity of the lithium-ion cell. Its deficiency (a too small amount) causes a loss of capacity and disrupts the cyclical operation of the cell. In turn, its excess causes a decrease in energy density. Optimization tests were carried out, consisting of wetting the individual elements of the cell, i.e., electrodes and the porator, and thus determining the necessary volume corresponding to the pore volume. Additionally, the excess VC (vinyl carbonate) was analyzed with the lack of electrolyte in the cyclic operation of the system. The mechanism and dependence were limited to the voltage changes in the cell—the lack of the drug contributed to its drop right at the first stage of discharge. In turn, too much VC also leads to a voltage drop, but much later—only at the end of the discharge process. The EIS technique is becoming useful in detecting electrolyte redistribution in the pores. Thanks to this analysis, we can determine the electrolyte decomposition after the complete wetting of the electrodes and separators and determine whether the used electrolyte volume was sufficient for the specific pore structure. All analyzed operating points of the cell have a significant impact on the internal resistance of the cell, defined as a function of the electrolyte volume used. The commercialization of lithium-ion cells forces scientists to carry out a detailed analysis of the composition and volume of the electrolyte used, especially when the electrode surface comes into contact with the active material.

Mixtures of an organic solvent and lithium salt are used as electrolytes in lithium-ion cells. The electrolyte solution must be capable of transporting lithium ions freely, which requires a high dielectric constant as well as a low viscosity. Most often, electrolytes are a mixture of two or three solvents and a lithium salt, because none of the solvents used alone meet the above-mentioned conditions. The main types of solvents used for electrolytes in lithium-ion cells are organic carbonates, lactones, ethers, sulfones and nitriles. Cyclic carbonates (most often PC, EC) show a high value of the dielectric constant, which improves the solubility of lithium salts with high viscosity due to strong intermolecular interactions, which, in turn, hinders the transport of ions (Table 2).

In contrast, linear carbonates such as DMC and DEC show lower permeability and lower viscosity due to the linear structure that increases the degree of freedom of the molecule. Therefore, mixtures of linear and cyclic carbonates are often used, e.g., the EC/DMC mixture (1:1 by weight), which has a positive effect on the size of the cell capacity. The lower viscosity of the latter is associated with its lower flash point, which raises safety concerns.

**Figure 3:**
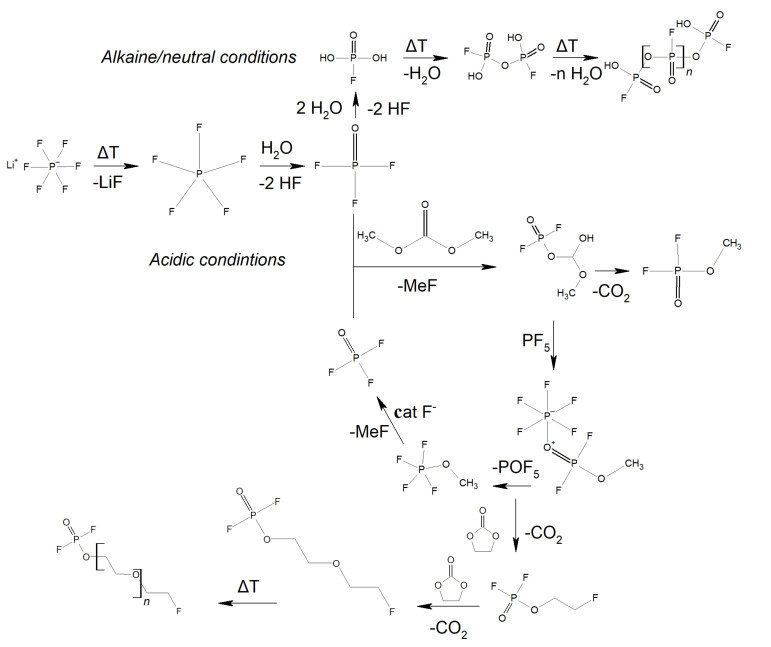
The mechanism of degradation of 1 M LIPF_6_ in EC:DMC.

The authors propose that, in the upper part of Figure 3, under neutral or basic conditions, the reaction between POF_3_ and H_2_O is allowed and, because this reaction is fast, in the first hours the POF(OH)_2_, POF_3_, and HF are produced [69]. Then, the acidity of the electrolyte increases, and all water is consumed. Now, POF(OH)_2_ polycondensates and POF_3_ attack the solvents and self-sustained reactions initiate. The thermal decomposition mechanism of LiPF_6_ is connected with the solvents used to dissolve the salt. For DMC-LiPF_6_, PF_5_, OPF_3_, CO_2_, Me_2_O, and OP(OMe)F_2_ are obtained, while for the DEC-LiPF_6_ system, PF_5_, OPF_3_, CO_2_, Et_2_O, EtF, OP(OEt)F_2_, and OP(OEt)_2_F are produced. The authors also show that preventing the transesterification of dialkyl carbonates should inhibit the thermal decomposition of the LiPF_6_/carbonate-based electrolytes [69,70].

**Figure 4:**
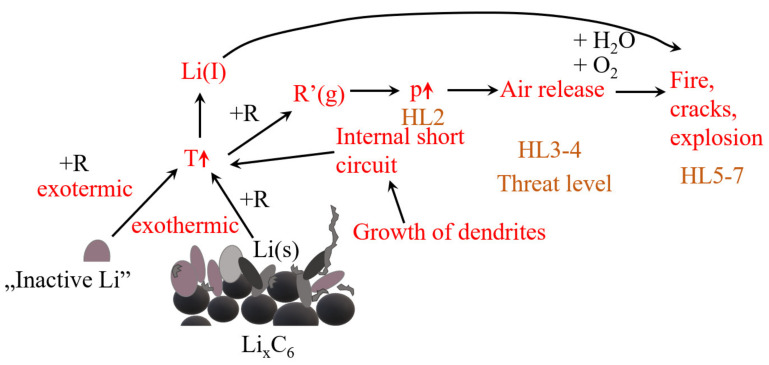
The effect of lithium deposition on the safety of LIBs, where HLs are the different hazard levels of degradation of 1 M LIPF_6_ in EC:DMC.

Lithium (Li) deposition occurs in commercial LIBs. It has a significant impact on the safety, life, fast-charging capability, and low-temperature performance of LIBs. This is usually due to the cell’s aging mechanisms. This process takes place as a reaction parallel to intercalation. The use of Li-ion cells outside of the specification or defects inside the cells can lead to a catastrophic failure (thermal runaway). Figure 4 presents the effect of lithium deposition on the safety parameters [69,71,72]. First, lithium dendrites grow from the anode surface through the separator leading to heat generation. Second, exothermic reactions of the deposited lithium lead to heat generation. Third, overcharging can lead to lithium deposition and to exothermic reactions due to the charging current. The authors [30] also showed that these processes could lead to a thermal runaway.

### 2.3. Non-Flammable Electrolytes

#### 2.3.1. Composite Polymer–Ceramic Electrolytes

An important aspect is that there is no spontaneous combustion reaction upon sudden heating. Therefore, heat control is important during the reaction of the electrolyte with the electrode material [1].

This solution overcomes the disadvantages of using the polymer and the ceramic electrolyte separately. Thanks to the appropriate synthesis, it is possible to get the expected ionic conductivity, prevent the formation of dendrites, and ensure an appropriate number of lithium-ion transfers. The advantages are high mechanical, chemical, and electrochemical resistance as well as high electrochemical oxidation potential. Such materials (divided into active and inactive) consist of a polymer matrix and a ceramic filler. Passive fillers include, e.g., zircon (IV) oxide, yttrium (IV) oxide, silicon oxide, titanium oxide, and aluminum oxide. In turn, active fillers are responsible for imparting the ionic conductivity. Of these, perovskite, sulfide electrolyte, and sodium (Na) super ionic conductors are distinguished. Ion transport is caused by the defects in the crystalline ceramic electrolytes. The disadvantages remain the low flexibility and high production costs on a large scale [73]. Table 3 presents some of the composite electrolytes and their ionic conductivity.

#### 2.3.2. Ionic Liquids

Ionic liquid is an ionic chemical compound composed of a cation and an anion. Room temperature ionic liquids (RTILs) are salts, which exhibit melting points below 100 °C. Ionic liquids are not a molten salt or an aqueous solution. They have different interesting properties, such as:

- Remaining liquids over a wide temperature range;
- Dissolving organic and inorganic compounds;
- Showing thermal and electrochemical stability;
- Being practically non-volatile;
- Having electrical conductivity;
- Dissolving catalysts (transition metal complexes);
- Keeping the activity of enzymes;
- Having catalytic action.

Ionic liquids are used as nonflammable electrolytes in LIBs (Table 4). These include Py_13_TFSI (N-propyl-N-methylpyrrolidine bis(trifluoromethanesulfonyl)amide) and MPPipTFSI (N-methyl-N-propylpiperidinium bis(trifluoromethanesulfonyl)imide). They show similar properties to conventional electrolytes and electrode materials. However, there are still some issues, that must be resolved, including low rate capability arising from their high viscosity, low Li transference number, redox reactions instability, and economic aspects [7]. When using ILs as electrolytes, suitable separators are needed so that the ionic liquid is capable of wetting it thoroughly. This reduces the internal resistance of the cell. In this case, the polymer must exhibit high chemical, thermal, and mechanical stability. Ceramic additives and appropriate optimization of the separator production process are essential. In this case, microporous separators (coated or novel commercial separators) or non-woven separators (electrospun PAN separators, PVDF-HFP separators, or those with novel additives) are used [84].

In [85], Qi et al. reported a high-voltage resistant ionic liquid for LIBs. 1-hexyl-1-methylpyrrolidinium bis(trifluoromethylsulfonyl)imide ([C_6_Py][TFSI]) exhibited the highest decomposition voltage at approximately 5.12 V. Ionic conductivities of LiFTSI [C_6_Py][TFSI]s with the increasing LiFTSI concentration (0.5–1.25 M) were in the range of 11 to 4.4 × 10^−2^ S cm^−1^ at 25 °C. The authors in [86] reported protic ionic liquids for LIBs (The electrolyte 1 M LiTFSI in triethylammonium bis(tetrafluoromethylsulfonyl)amide Et3NHTFSI). The cell exhibited small capacities (during the test at 0.1 C and 10 C the LIBs delivered a capacity of 115 mAh g^−1^ and ~30 mAh g^−1^, respectively), which must be improved. In 2015 [87], a prototype of the cell with a bis(fluorosulfonyl)imide (FSI)-based ionic liquid electrolyte was developed and used in the extreme environment of space. The cells were evaluated for radiation and vacuum tolerance. The prototypes passed all the tests and exhibited no deterioration in high vacuum while exhibiting high-performance without rigid housing or potting. Ionic liquids, such as FRs, show high efficiency during combustion, which affects their properties that protect the devices against heating [14]. Also, ILs are used as an additive to conventional electrolytes. Using a bottom-up approach, it is possible to design a novel dicationic ionic liquid as an additive to conventional EC+DMC solvents for LIBs. In [88], the authors showed that the cell with the ionic liquid-additive showed better specific capacity and coulombic efficiency (as much as 99% after 100 cycles) compared to the conventional solution.

**Table 4:** Structures of different ionic liquids used to reduce the flammability of LIBs.

Name	Abbreviation	Structure	References
Cation dialkyl imidazolium and anions	-	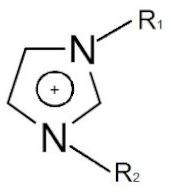	[89]
		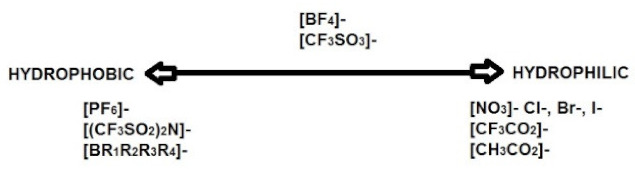	
1-Allyl-3-methylimidazolium bis(trifluoromethanesulfonyl)imide	AMIMTFSI	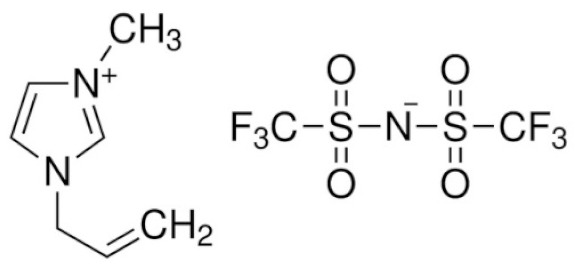	[90]
N-n-butyl-N-methylpyrrolidinium hexafluorophosphate	([Py14]PF_6_)	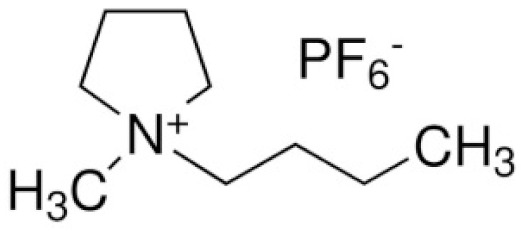	[91]
1-Ethyl-1-methyl piperidinium bis(trifluoromethanesulfonyl)imide	EMP-TFSI	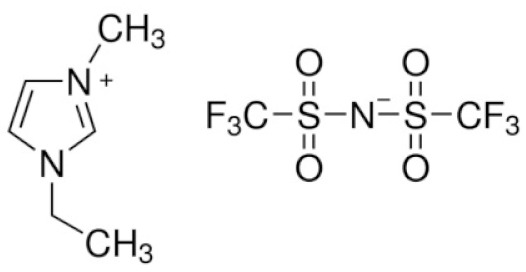	[92]
1-butyl-3-methyl-imidazolium tetrafluoroborate	BMIMBF4	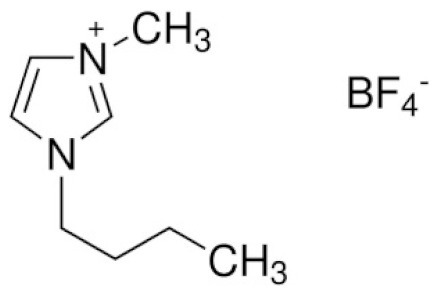	[93]
N-methyl-N-propylpiperidinium bis(trifluoromethanesulfonyl)imide	PP13TFSI	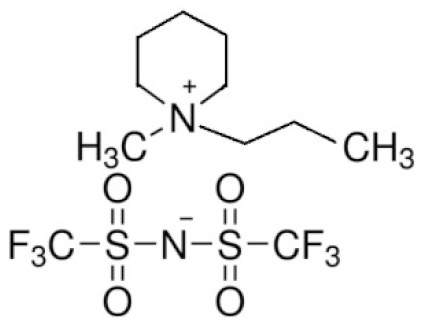	[94]

#### 2.3.3. Polymeric Ionic Liquids

Polymeric ionic liquids (PILs) are formed when IL molecules are joined to polymer chains. They are also referred to in the literature as polymerized ionic liquids or poly (ionic) liquids. The nature of the cation as well as the anion affects the mobility of the ions in the PIL. Additionally, the molecular weight, the nature of the polymer chain, and the molecular weight of the polymer are also important, as well as other variables such as PIL moisture [11].

#### 2.3.4. Polymer Electrolytes

Polymer electrolyte (Table 5) contains an MX salt (the cation and anion are mobile ions), while in a polyelectrolyte the anion is covalently bonded to the polymer network and the H+ counterion acts as a mobile (conductive) cation. The conductivity in polymer electrolytes is located between the conductivity in liquids and defective crystals (based on vacancies, defects, and holes). In addition to the polymer backbone, they also contain ions that are not bound to the polymer backbone and sometimes not even to the solvent molecules. Ionic conductivity occurs in amorphous regions of the structure. In polymer electrolytes, the ion transport mechanism occurs in the following two ways:

- Void space theory (as a result of thermal changes, voids are formed, in which other ions may lodge);
- Percolation (leakage) theory (ion conductor as a system consisting of a series of conductive islands, around which there is a non-conductive area).

They show such properties as increased stability, ionic conductivity, energy density, and low volatility, and they use no solvents, have low weight, are easy to form, and are safe in relation to conventional electrolytes. Conductivity in the amorphous region is also an advantage, which reduces the cost of preparing the electrolyte to achieve a crystalline structure, as is the case with electrode materials. Ion conductivity is related to the donor and acceptor according to the Lewis theory. They are a good option to prevent the formation of lithium dendrites. In lithium-ion cells, they act as a separator and electrolyte, which significantly reduces the costs associated with the lack of an additional separator or solvent for the electrolyte. Due to their dual function, they must meet the following requirements: high ion conductivity (above 10^−4^ S cm^−1^ at room temperature), high electronic conductivity (above 10^−6^ S cm^−1^), high ion transfer number, and high operating temperature range while being in line with the idea of green chemistry. An important factor is the glass transition temperature, which must also be sufficiently low to prevent changes in the component structure. Despite many advantages, the electronic conductivity is significantly lowered and requires an improvement in order to be used on a larger scale [95].

**Table 5:** Polymer electrolytes used in lithium-ion cells.

Cell	Discharge Specific Capacity/mAh g^−1^	References
Li/poli (bisAEA4-0.4 M)	162 at C/20	[96]
LiTFSI-MPPipTFSI)/LiFePO_4_	134 at C/2	
Li/80% LiNfO-EMImNfO + 20% PVDF-HFP/LiCoO_2_	164 (C/10)	[97]
Li/P(VdF-HFP)-LiTFSI-PYR_14_	145.77 (at 60 °C)	[98]
TFSI (1:1:1)/LiFePO_4_	158.75 (at 80 °C)	
Li/PVdF/LiCoO_2_	149.1	
Li/PVdF + SiO_2_/LiCoO_2_	152	[99]
Li/PVdF + (SiO_2_-PAALi)/LiCoO_2_	156.5	

**Figure 5:**
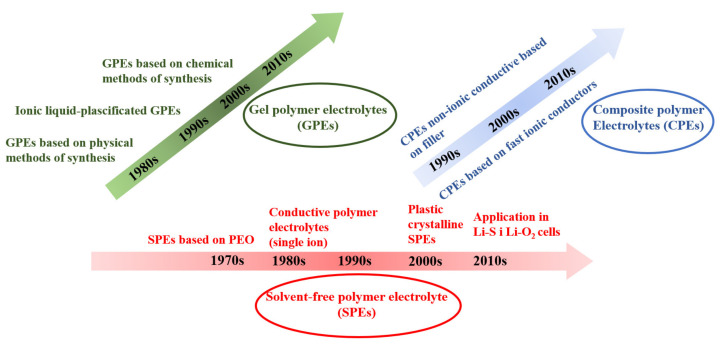
Chronological development of polymer electrolytes for non-aqueous lithium-based cells in the years 1970–2010.

Polymer electrolytes for Li-based batteries can be divided into three major categories: solvent-free polymer electrolytes (SPEs), gel polymer electrolytes (GPEs), and composite polymer electrolytes (CPEs) (Figure 5) [100]. An important group of polymer electrolytes are plasticized polymer ones exhibiting high conductivity, while their disadvantage is low mechanical and chemical stability [101].

#### 2.3.5. Gel Polymer Electrolytes (GPEs)

GPEs are composed of polymer matrices, liquid solvents as plasticizers, Li salts, and additives such as inorganic fillers [102]. High cost and poor mechanical strength are still the key barriers for the application of GPEs in large-scale batteries. Different methods are used to prepare GPEs. The non-porous GPEs (PEO-, PAN-, PMMA (poly(methyl methacrylate))-, and PVDF) are synthesized via casting (dissolution of a polymer matrix and membrane casting on the flat substrate), in situ polymerization, dip coating, hot-press, and screen printing techniques. To prepare the porous GPEs (to improve the absorption of the GPEs liquid electrolyte, e.g., P(VDF-HFP)-based GPE), the Bellcore method (liquid extraction), the phase separation/inversion method, electrospinning, and foaming technologies are applied. In order to modify the GPEs by adding inorganic nanoparticles, blending, crosslinking, copolymerization, and composite formation are utilized [103]. Also, dual-functional novel PETEA-based GPEs (LiNi_0.8_Co_0.15_Al_0.05_O_2_(NCA)/graphite GPE and NCA/graphite–Si/C GPE) have been reported with a superior rate, much lower gas generation, higher life cycle, and improved safety performance [104] along with a capacity retention after 200 cycles (at the discharge rate of 5 C) of 92.5% and 81.2%, respectively. In [105], the synthesis of GPEs with graphene oxide used as a filler was applied. Here, the discharge specific capacity retention rate of the battery (LiFePO_4_/Li) was 94.4% after 100 cycles at 0.2 C. This application also increased the safety of the LIB and lithium-ion transference number up to 0.79. In [106], Li et al. presented the asymmetric GPE with high Li^+^ conductivity. This system enables a reduction of dendrites. The poly(vinylidene fluoride-co-hexafluoropropylene) (PVDF-HFP) in the Li|LiFePO_4_ system can deliver the coulombic efficiency of 99.5% at 2 C after 600 cycles. An interesting idea has been presented by Logan et al. in [107]. They proposed the UV-cured eutectic GPE to make LIBs safer and more robust. When testing the Li_4_Ti_5_O_12_/LiMn_2_O_4_ (lithium manganese oxide (LMO)) full cells, those that contained GPEs at 0.45 C showed the coulombic efficiency of as much as 100% after 200 cycles during the charging process.

GPEs are used in LIBs to enhance mechanical properties (ionic-conductive inorganics), ameliorate interfacial stability (inner nanofillers, e.g., graphene oxide), improve ionic conductivity (plasticizers, e.g., ionic liquids), and increase thermal stability (e.g., cellulose, MOFs (metal organic frameworks)) [108]. In [109], the authors presented the CPE for a high-performance lithium-ion battery. This polymer consists of polyethylene-glycol dimethyl-ether (MW 2000 g mol^−1^), lithium bis(trifluoromethanesulfonyl)imide (LiTFSI) conducting salt, lithium nitrate (LiNO_3_) film-forming additive, and a nanometric silica (SiO_2_) filler. The electrolyte has a conductivity over 10^−4^ S cm^−1^ above 45 °C and a high stability. A polymer in the Li/LiFePO_4_ cell at 50 °C stably delivers the capacity of 150 mAh g^−1^.

**Figure 6:**
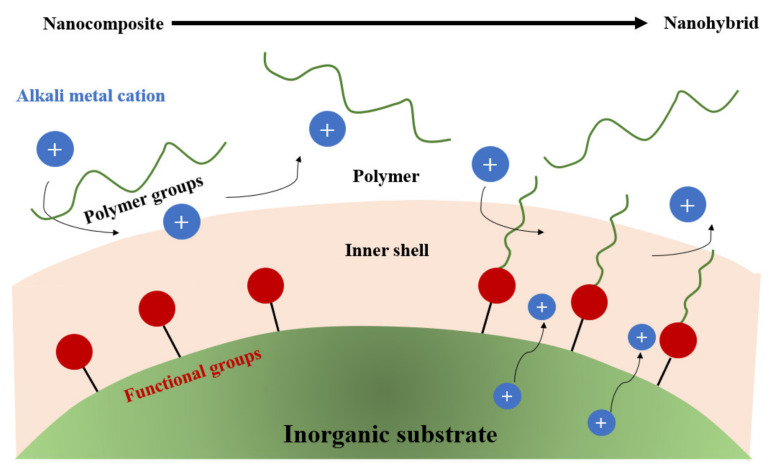
Challenges and opportunities for CPEs and HPEs [102].

Figure 6 shows the structures of CPEs and HPEs (hybrid polymer electrolytes) and the structure challenges connected with their applications. HPEs (e.g., hybrid systems with poly (ethylene glycol) (PEG)), herein refer to the electrolyte materials where the organic and inorganic components are bonded together via strong chemical interactions. They are dry electrolytes where we can distinguish a composite polymer electrolyte with inactive/active filler, a solid polymer electrolyte, and an inorganic solid electrolyte. Unfortunately, they are far from meeting expectations due to various automatic applications (ionic conductivity, lithium transference number, electrochemical stability, and mechanical modulus). However, upon overcoming these issues, CPEs and HPEs would certainly become future-oriented electrolytes for development in LIB technology [102].

#### 2.3.6. Solid Polymer Electrolytes

GPEs and SPEs (also called solvent-free polymer electrolytes) generally consist exclusively of polymer matrices and Li salts as solutes without the addition of liquid solvents as plasticizers [110]. They have to meet such expectations as, for example, cation solvation nature, dielectric constant, backbone flexibility, and high molecular weight. There are different kinds of SPEs [102]. These are polyethylene-oxide-based (P(EO)_6_-LiAsF_6_), polycarbonate-based (cellulose-supported poly(propylene carbonate)-based), polyester-based (copolymer of trimethylene carbonate and caprolactone) and polysiloxane-based (poly(siloxane-g-ethylene oxide)-based) SPEs. In [111], the authors create a high-voltage SPE based on a star-comb PDLLA-PEG (poly(d,L-lactide)-poly(ethylene glycol)methyl ether methacrylate) copolymer for LIBs. PDLLA-SPEs exhibits good thermal stability up to 270 °C and in the range −48 to −34 °C as well as optimal ionic conductivities of 9.7 × 10^−5^ S cm^−1^ at 60 °C. In [112], He et al. demonstrate a highly conductive solvent-free polymer electrolyte membrane for lithium-ion batteries combined with poly(ethylene glycol)diacrylate prepolymer, LiTFSI, and succinonitrile plasticizer that obtained ionic conductivity at an ambient temperature equal to ~1.4 × 10^−3^ S cm^−1^ along with excellent electrochemical stability (4.8 V vs. Li/Li^+^) as well as thermal stability up to 140 °C.

They show such properties as flexibility, long life, low weight, high energy density, and thermal resistance, and are solvent-free and have the potential for miniaturization [113]. Am important aspect of SPEs is their thermal stability. PEO-PMMA-LiClO_4_, with a PA plasticizer and NMP as solvent, shows an ionic conductivity of 1.59 × 10^−5^ S cm^−1^ and thermal stability up to 209 °C [114], while PAN-PVA-LiClO_4_ with EC/DMC as a filler and EC/DMC as solvent presents an ionic conductivity of 2.5 × 10^−4^ S cm^−1^ and is thermally stable up to 300 °C [115]. The best properties are exhibited by PVA (polyvinyl alcohol)–aluminum ammonium sulphate 12 aqueous and water Pas a solvent because of its high ionic conductivity of 1.73 × 10^−4^ S cm^−1^ and thermal stability up to 350 °C [116].

### 2.4. Commonly Used Flame Tests

#### 2.4.1. Self-Extinguishing Time (SET) Method

To better understand the flame-retardant function, the self-extinguishing time (SET) method is used. The time could be defined as follows:

(1) $$SET=\frac{t}{m}$$

where *t* is the time needed for the combustion process of electrolyte from ignition to extinguishment and *m* is the mass of the electrolyte (s g^−1^). The smaller the SET, the more stable the electrolyte. There are three ranges: 6 s g^−1^ (nonflammable electrolyte), 6 s g^−1^ to 20 s g^−1^ (less flammable electrolyte), and higher than 20 s g^−1^ (flammable electrolyte) [5].

#### 2.4.2. Flash Point (FP) Method

The flash point is defined as the lowest temperature at which a liquid generates flammable vapors that can be ignited in the air by a flame above its surface. The most common are the Abel and Pensky–Martens closed-cup methods (standard norms, because it is not a physicochemical parameter) [117]. Closed-cup methods are used in a battery pack or motor compartment, while open-cup methods are utilized in an open environment. All standardized methods with descriptions have been presented in Table 6 [117].

To calculate the FP, the formula given in Equation (2) is used:

(2) $$T_{F}=a+{bT}_{B}+cT_{B}^{2}$$

where the authors in [118] have shown the correlation between (closed-up) FPS (*T_F_*) of organic substances and their BPs (*T_B_*). Both temperatures are given in K and the coefficients *a*, *b*, and c are obtained from a linear regression analysis where *T_F_*/*T_B_* is known.

#### 2.4.3. Thermogravimetric Analysis (TG) Flash Point (FP) Method

TG is commonly used to examine the stability of different samples. The changes in the percentage loss of mass as a function of time or temperature could be easily observed. It is known that, the smaller the loss of mass during heating, the more stable the sample. In order to determine the FP, the difference is applied between the experimentally determined FP (using automatic FP testers) and the selected temperatures of decomposition (T_sd_).

#### 2.4.4. Differential Scanning Calorimeter (DSC)

The DSC method is used most frequently in the examination of polymer probes to highlight different processes that occur during heating and cooling (crystallization, glass transition) and, as a result, we may also obtain thermodynamic heat of the corresponding transformations. Here, the FP values usually locate during the first decomposition step of the sample [119].

#### 2.4.5. Accelerating Rate Calorimetry (ARC)

The ARC method is used to study the thermal reactions in the electrolytes of lithium-ion cells [120]. First, the solutions are heated to the appropriate temperature at a given temperature increment per minute. Then, a self-heating of the electrolyte occurs with an appropriate sensitivity threshold.

Table 7 presents the advantages and disadvantages of the commonly used methods that allow for estimation of the flammability of the electrolyte [117].

A thermostat is used to maintain the temperature for a specified period of time to achieve the equilibrium between the sample and the calorimeter. After the measurement, the device is chilled with liquid nitrogen and gases are released through the valves. The results are presented in the following system: temperature (°C)–self-heating rate (°C min^−1^)–pressure developing rate (psi min^−1^) [121,122].

### 2.5. Flammability of Different Electrochemical Systems

Fire risk is a combination of fire hazards and the probability of occurrence thereof. A fire hazard is defined as a “potential for fire-related harm” [123]. Fires can be divided as occurring in five groups: (1) flammable liquids (e.g., petroleum lubricants), (2) common flammable materials (e.g., wood), (3) flammable metals (e.g., magnesium, lithium), (4) kitchen appliances (flammable agents), and (5) electrical appliances connected to a source of electricity [14]. The heat release rate (HRR) is one of the most important parameters that define a fire hazard. The combustion process has four stages: heating to ignition, violent ejecting or explosion, stable burning/weakening, and extinguishment [124]. During the normal cycling within the designed voltage range, the gas is generated mainly due to ester exchange reactions (CO_2_, CO, CH_4_, C_2_H_4_, C_2_H_6_, C_3_H_6_, and C_3_H_8_). When a cell gets heated above 130–150 °C, exothermic reactions between the electrodes and electrolyte set in, increasing its internal temperature and, if more heat is generated than dissipated, the fire can occur [21]. There are various factors influencing the thermal stability of LIBs: aging, state-of-charge (SOC), or positive active materials. Also, internal heat, crush, intrusion (nail penetration), internal short-circuit (dendrites), external short-circuit, and external heat (thermal propagation) influence the thermal stability of the system. To prevent a fire, different methods are used [125]:

- Inherent safety methods (cathode modification, anode modification, safe electrolyte);
- Safety devices (safety vents and current interrupt devices, positive temperature coefficient devices, shutdown and ceramic-coated separators, battery management systems (BMS));
- Fire suppression and cooling (fire detection, fire extinguishing agents and methods).

**Figure 7:**
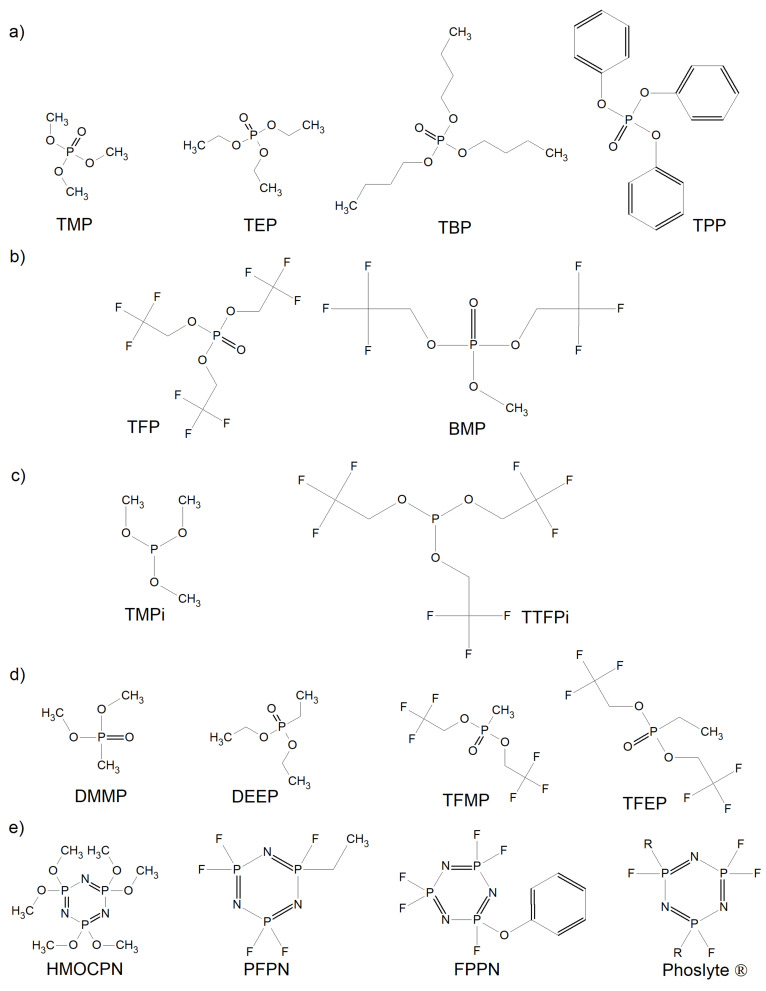
Molecular structures of (**a**) phosphates; (**b**) fluorinated phosphates; (**c**) phosphites; (**d**) phosphonates; and (**e**) cyclophosphazenes used as flame retardants in LIBs.

Most common flame-retardants may be divided into two categories according to their mechanism of flame-retarding: condensed-phase and gas-phase [6]. Generally, due to their construction, they are divided into ionic (e.g., TFSI), composite (e.g., ethoxy(pentafluoro)cyclotriphosphazene PFPN), phosphorus (e.g., triphenyl phosphate TPP), and fluoride (e.g., methyl nonafluorobutyl ether (MFE)) liquids. They can increase the flash point of the electrolyte, making it less flammable. Figure 7 presents flame-retardant additives used to overcome stage 3 of the thermal runaway process [126]. The chemical radical scavenging process describes the flame retardant action of phosphorus-containing compounds During combustion, phosphorus-containing molecules can break down phosphorus-containing free radicals which terminate the radicals (OH and H radicals) generated during chain propagation and can lead to continuous combustion. In fluoride compounds, the fluoride substituents are flame retardant. This can be compared to Teflon (poly(tetrafluoro)ethylene) that prevents high-temperature ignition in Teflon pans/pots. Composite additives are applied to reduce the amount of one agent, while ensuring adequate solubility and electrolyte compatibility. It is important that the applied flame retardant does not significantly affect the capacity retention and charge/discharge capacity of the cell.

It is also important that flame retardants meet certain expectations such as good chemical stability, electrochemical inertia, suitable physical properties (conductivity, boiling point, viscosity), low toxicity, low cost, and good machinability [5].

**Figure 8:**
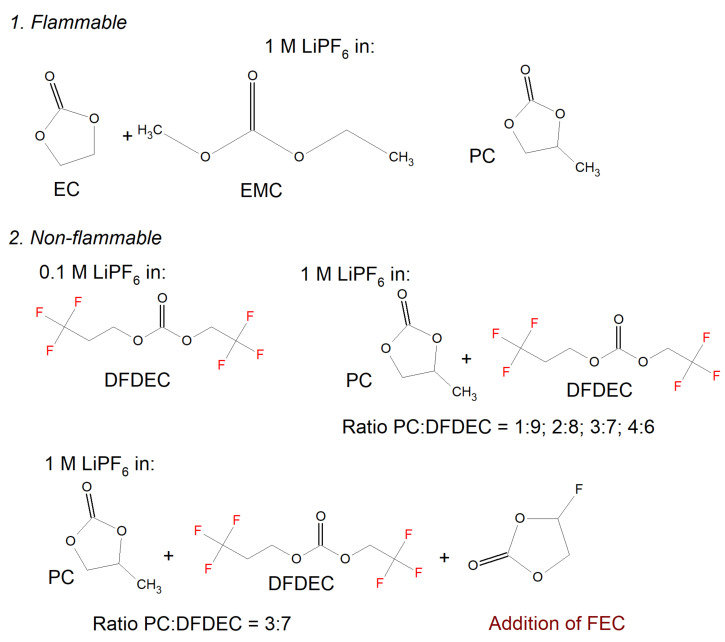
Flammability test results for (**1**) LiPF_6_/EC conventional electrolytes: DMC (ratio 3:7 vol); 1 M LiPF_6_/PC; (**2**) 0.1 M LiPF_6_/DFDEC; 1 M LiPF_6_/PC: DFDEC in volumetric ratios of 1:9, 2:8, 3:7, 4:6; 1 M LiPF_6_/PC: DFDEC (3:7) with an addition of 1 wt.% FEC.

Figure 8 demonstrates the flammability test results for some systems using different solvents and additives [127]. It shows that DFDEC (di-(2,2,2-trifluoroethyl) carbonate), PC (propylene carbonate), and FEC (fluoro-ethylene carbonate) have a beneficial impact on the system flammability, while electrolytes utilizing classic EC, EMC (ethyl methyl carbonate) solvents are still flammable [128]. 0.6Li_2_MnO_3_· 0.4LiNi_0.45_Co_0.25_Mn_0.3_O_2_ with 5% of DFDEC in 1 M LiPF_6_ in EC:DMC reached a high capacity of 250 mAh g^−1^ with an excellent charge–discharge cycling stability at 0.2 C [128].

The flammability of different electrolytes and applied tests have been presented in Table 8. So it would be possible to ensure the appropriate thermal resistance of the electrolyte, flame retardants were collected and methods of monitoring the flammability by using various mathematical and thermodynamic models were indicated.

Unfortunately, flame retardation does have side effects, mainly when it comes to electrochemical performance. To improve this trade-off, some modifications of structures are required, e.g., use of compounds that have both film-forming and flame-retarding properties (stable SEI forms) [7]. In Table 9, literature data describe different electrochemical systems (2001–2020) and flame test methods. The most commonly used methods of checking the flammability of the system are the SET test, flammability test, determination of flash point, DSC, TG, and ARC.

**Figure 9:**
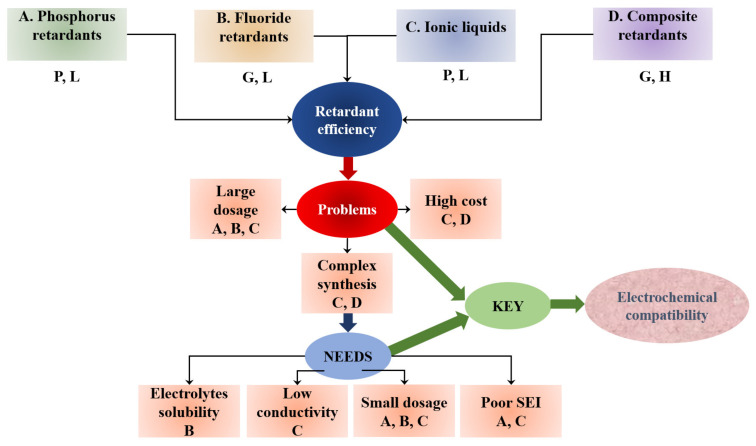
Summary selection of various electrolyte retardant additives, where A, B, C, and D mean the type of flame retardant used. P—poor electrochemical compatibility, G—good electrochemical compatibility, L—low retardant efficiency, H—high retardant efficiency.

Figure 9 shows the key balance between electrochemical performance and retardant efficiency. Using the synergic effect between various flame retardant additives allows the dosage to be reduced, leads to a decrease in the cost, and improves the electrochemical parameters [5]. It should be noted that ignition of one cell may lead to the spread of fire to another. Such a module is capable of generating much more heat than a single cell, which also produces more toxic gases when burned [212]. Apart from the discussed methods of protection against electrolyte flammability, self-cooling electrolytes are also used [213]. The authors propose a composite self-cooling electrolyte that reduces flammability without compromising the electrochemical performance. PFMP acts as a flame retardant and internal extinguishing agent.

## 3. Hydrogen—Future

Fuel cells, also known as hydrogen cells, are electrochemical devices. The classification of fuel cells is mainly based on the specific electrolyte. A distinction is made between the following fuel cells: alkaline electrolyte cell, PEMFC capillary cell, polymer electrolyte cell, direct methanol cell, solid oxide cell, molten carbonate cell, phosphoric acid cell, formic acid cell, carbon fuel, and microbial cell. Another, additional classification is a division according to the cell temperature. This type of cell can be used in portable devices such as laptops, cameras, and smartphones, i.e., those that have low-power batteries. They work very well in transport, preferably as an additional source of power in hybrid vehicles. This type of power source has many advantages. It does not generate unpleasant noise or vibrations and does not consume hydrogen when stationary, as is the case with internal combustion engines.

This issue was raised at the end of this article due to the solutions regarding fuel cells and various electrolytes. Their mixing with non-flammable additives has not been described in the literature, because these cells were quickly commercialized. We believe that this thread is worth mentioning in light of the dynamic, rapidly developing automotive industry.

### 3.1. Introduction

Fuel cells are being increasingly discussed because of their invaluable advantages, which mainly include their high efficiency and harmlessness to the environment. The application areas of fuel cells are very wide owing to the possibility of their use wherever there is a need to generate electricity and heat. We are witnessing a dynamic development of unconventional energy areas, in which technologies based on hydrogen as fuel play a significant role. The rivalry between financial giants, whose main interest is new energy technologies for the future, is the driving force behind this research. It is obvious that development and progress depend on an abundance of electricity produced from renewable sources with maximum environmental protection. Despite the fact that the modern market of fuel cells is still very limited, there are an enormous number of technological solutions for devices to be designed in the future or already in the market. There are four main types of cells named after the electrolyte applied in them: phosphoric acid, molten carbonate, solid oxide, or proton exchange membrane.

**Figure 10:**
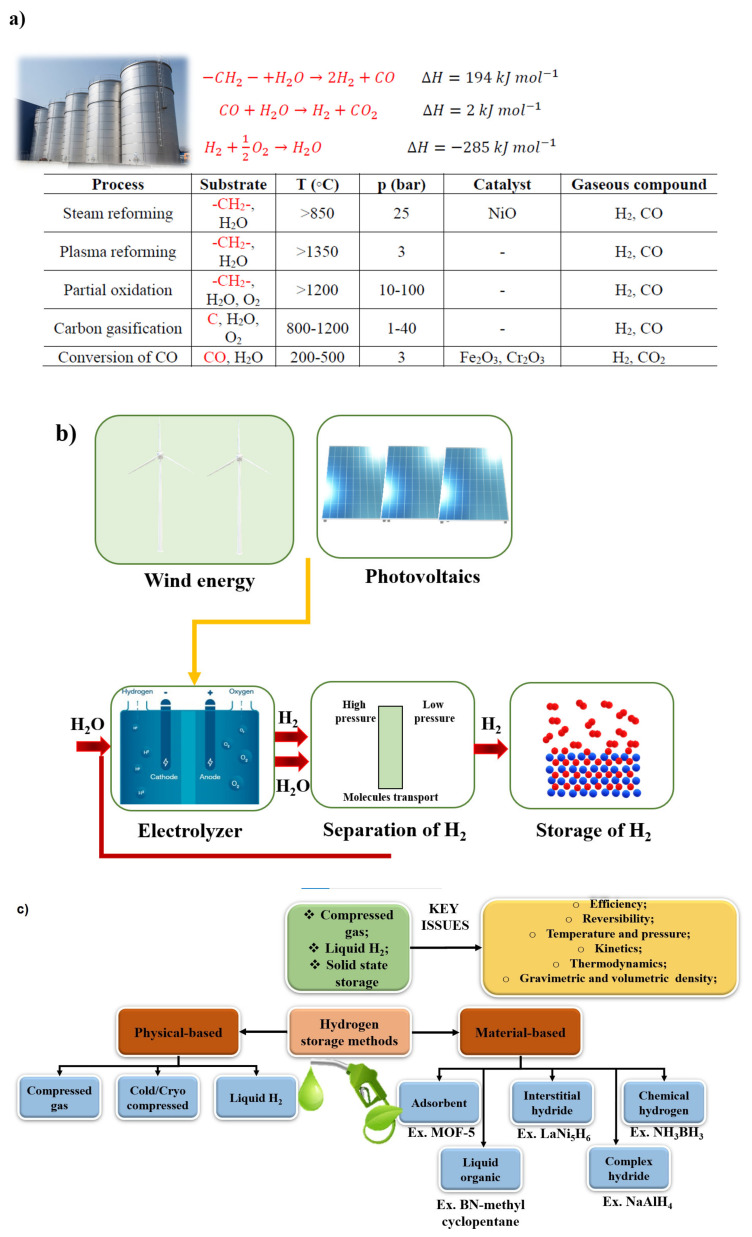
Hydrogen: how to obtain (**a**,**b**) and how to store (**c**).

One of the most promising applications for hydrogen is transport. Transport is one of the main sources of environmental pollution on a global scale, resulting in global warming caused by the greenhouse effect and local warming manifested by the presence of smog in urban agglomerations. The problem of emissivity of vehicles of various categories has been discussed in many scientific papers [214,215,216,217,218]. In addition, the number of vehicles worldwide is projected to double by 2050 [219]. This justifies the need for research and development into low-emission vehicle solutions. Zero-emission buses, regional trains, shunting locomotives, and taxi fleets will provide a motivation for increasing hydrogen production and expanding infrastructure. The most popular methods of obtaining hydrogen and its storage are presented in the Figure 10.

When it comes to water-splitting, we can distinguish four main methods: photolysis, nuclear energy processes, electrolysis, and thermolysis. Electrolysis is a process by which a constant electric current breaks a chemical bond between hydrogen and oxygen in an aqueous solution: 2 H_2_O → 2 H_2_ + O_2_.

Very pure hydrogen gas is in turn formed at the cathode, from where it is discharged and then stocked. The formation of oxygen at the anode also cannot be ignored, without which it would a given technology may not work because it cannot react on just one electrode. The process can also be carried out at room temperature and only requires electricity as energy. The performance of current commercial electrolyzers used to produce hydrogen is about 50–75%.

The principle of operation of the cells is based on the electrochemical reaction of hydrogen with oxygen, during which energy and heat are generated, and the only by-product is water. In cars, the whole process begins with the supply of hydrogen from the high-pressure tank to the cell. Compressed air is also supplied in parallel. The reaction in the cell produces a current that is converted into an alternating current and supplied to the electric motor responsible for traction.

For the electrolysis process to take place, the external voltage of the power source must be higher than the EMF of the cell in which the reverse reaction takes place in the electrolyzer. These factors determine what reactions will occur at the electrodes as the current flows through; at the cathode, they first discharge heavy metal cations. If the electrolyte solution does not contain heavy metal ions, hydrogen is released at the cathode from discharging H^+^ cations or reducing water molecules. In the case of an acidified solution heavy metal salt, the evolution of metal and hydrogen can occur simultaneously. At the anode, the anions of the anaerobic acids are discharged first. If they are absent in the solution, oxygen is released at the anode from the discharge of OH^−^ or ions’ oxidation of water molecules. Unfortunately, the efficiency of hydrogen production using the water electrolysis process is very low, despite the high purity of the obtained hydrogen, in terms of being economically competitive. Thus, in order to increase efficiency and reduce energy consumption, many researchers have done work to develop alternative methods, reducing the price of electrocatalysts, increasing efficiency, and reducing energy. Thus, when it comes to parameters that have to be improved, we can distinguish low durability, commercialization, acidic environment, and expensive components. Thus, we can combine different methods with electrolysis to overcome this, biological or enzymatic ones, which are connected with the green chemistry aspect.

In the production, other than the electrolysis of hydrogen, we can distinguish the following:

• Steam methane reforming (SMR)

SMR is a process in which natural gas or methane-containing streams (biogas or landfill gas) can react with water vapor in the presence of a catalyst, the products of which produce hydrogen and carbon dioxide. As a result of this process, a significant amount of hydrogen is obtained (70–75%). It is favorable technique, because of economic aspects, but unfortunately it is not very environmentally friendly because of carbon dioxide emission.

• Partial oxidation (POX)-gasification

In this method, the hydrogen is obtained using hydrocarbon fuels (e.g., coal, refinery products). In this process, substrate reacts with oxygen in a non-stoichiometric ratio and at high temperature. The product contains a mixture of carbon monoxide and hydrogen. The disadvantage is lower efficiency compared to the SMR technique, but by using a catalyst it is possible to lower the process temperature.

• Auto-thermal reforming of oil (ATR)

In this process, the heat needed for steam reforming in the catalytic zone is obtained from POX. Thus, the process is energetically and thermodynamically favorable. The advantage is a possibility to stop and resume the process. An important requirement is to have an appropriate ratio of both steam to carbon and oxygen to fuel.

• Biomass processing

In this processing, we distinguish thermochemical and biochemical processes. In thermochemical processes, we obtain higher reaction rates (high temperatures and cheap); pyrolysis (heating without oxygen) is included in this process. Here, it is possible to obtain a high amount of “synthesis gas” (a mixture of hydrogen and carbon monoxide).

• Biological process

This technology is used to produce a biohydrogen from biological materials. Here, we can involve algae, fermentation, and photogeneration. Enzymes also play a very important role. The biggest advantages are low initial investment costs and low energy demand, but unfortunately it has a very low performance, which can, however, be improved by coupling methods.

### 3.2. Road Transport and Hydrogen Fuel Cells

Statistically, almost 77% of road freight transport in the European Union is carried out by heavy-duty goods vehicles, which confirms that this type of vehicle is an essential element of the logistic chain of the European transport system. According to the Transport and Environment Report, in 2017 HDVs (heavy- duty vehicles) accounted for only 5% of all European vehicles and, at the same time, accounted for 26% of the CO_2_ emissions. Therefore, the idea of a “green supply chain” was created, which, according to one of many definitions, denotes management related to a full cycle of design, production, packaging, sales, use, and recycling, including the processes of storage, transport, and information exchange meeting relevant environmental standards [220]. It also refers to the concept of responsible supply chains and the idea of corporate social responsibility (CSR). The green supply chain, therefore, implies low- or zero-emission HDVs, which is why the use of hydrogen in long-distance road transport, particularly in truck transport, has great potential, though it requires significant investments in the development of the infrastructure and incentives schemes for fleet users. This particularly applies to companies serving international traffic, where hydrogen eliminates the barrier of short-range and long-time charging of electric vehicles.

The number of hydrogen-powered passenger cars is small, which is the result of a limited network of fueling stations, a small range of available vehicles, and their high purchase prices globally and locally in the European Union member states. The range of mass-produced, hydrogen-powered passenger vehicles is limited to just a few manufacturers and models [221].

### 3.3. The Bus and Hydrogen Fuel Cells

Buses fitted with hydrogen fuel cells are types of electric vehicles, but unlike traditional electric vehicles, they do not store energy in batteries but use hydrogen as fuel. The engine therefore uses the electricity generated in the fuel cell. As already mentioned, a fuel cell is designed to convert chemical energy from hydrogen into electricity. Furthermore, hydrogen-powered buses are equipped with a small battery that improves the vehicle performance and allows recuperation of energy from braking. Fuel tanks located on the roof of the vehicle store compressed hydrogen, which is the fuel used in the fuel cell. Hydrogen can be supplied through an appropriate generator installed within the hydrogen charging station or by transporting liquid or compressed hydrogen from the station [221]. Table 10 shows the pros and cons of hydrogen propulsion for bus transport.

### 3.4. Railway Transport and Hydrogen Fuel Cells

The basic structural elements of the railway vehicle fuel cell propulsion system are the fuel cell, fuel tank, traction engine, batteries, and main and auxiliary generators. The traction engine uses electricity generated in the fuel cell obtained either directly or from a reservoir. The accumulator should be properly adjusted by the main converter and its task is to create the tractive or braking force of the vehicle. The main converter also collects the energy generated during recuperation, which is subsequently transferred to the auxiliary converters and batteries. Auxiliary converters route the received electricity to various types of on-board devices such as air conditioning. Batteries store the electricity produced by the fuel cell.

**Figure 11:**
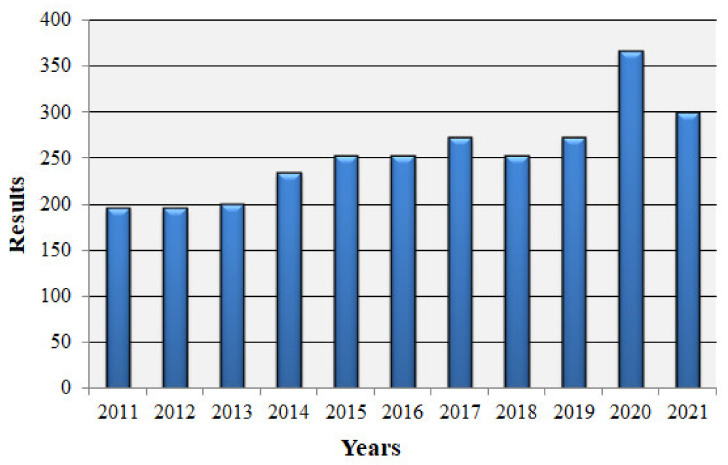
Graph of publications on hydrogen fuel cells vs. years (based on last 10 years of publications).

We observed the greatest increase in interest in hydrogen cells in 2020, which translates into the technology market (Figure 11). This analysis shows that the subject of hydrogen fuel cells is of great interest to scientists. Therefore, this article provides an overview of current trends in advanced fuel cell technology as well as their institutions.

They can also receive electricity from the recovery of energy generated during recuperation. The fuel tanks are located on the roof of the vehicle, where compressed hydrogen is stored. Table 11 shows the advantages and disadvantages of hydrogen propulsion in rail transport.

## 4. Conclusions

The aim of this work was to conduct a literature review of the systems used as electrolytes in lithium-ion cells and cells characterized by temperature ignition within the range of 90–150 °C, which allows them to be classified as non-flammable. Lithium-ion cells are widely used in industry and many of its branches, mainly in the production of electronic equipment such as laptops, mobile phones, or other portable devices. Technological progress and increasingly stricter requirements on safety in the production process and the subsequent proper use of the products by consumers have resulted in countless studies of the layouts that will provide the most comfortable use from the point of view of safety. The risk of ignition of the electrolyte used in the cell as a result of the high temperature generated due to the operation of the equipment is certainly small, e.g., in the case of telephone cells, but the risk is significantly increased with the use of such an electrolyte in a cell operating in a motor with a hybrid drive. Where people are the direct users of the equipment, this risk should be reduced practically to zero, hence the active involvement of researchers in searching for better and safer solutions. Tests have been conducted to find the right additives to the electrolyte so as to significantly shift the degree of the flammability limit while maintaining good values of others parameters determining the efficiency of the cell, its efficiency, and its durability.

The perfect electrolyte for a lithium-ion cell should have high ionic conductivity and a correspondingly high transfer number of lithium ions. Additionally, it should have high thermal stability, should not react with the electrode material or the material of the separator, and should meet the basic security requirements.

Carbon electrodes and lithium are very unstable and reactive systems in practice for all electrolytes used. Solutions that contain carbonates (EC, DMC, etc.) at elevated temperatures react violently with intercalated graphite lithium and cathodes, causing exothermic reactions. It is dangerous because most of the solvent mixtures used have a low boiling point and a flash point of 30–50 °C. Therefore, safety considerations have prompted work on new electrolyte systems that can be put into practice and which will significantly shift the flammability limit, such as those with polymer electrolytes or electrolytes containing an ionic liquid.

Ensuring safety in lithium-ion cells turns out to be a large challenge due to the existence of places susceptible to overheating in the cell and improper use of the cell, e.g., by overcharging or using the wrong currents. Exothermic reactions give rise to the domino effect: abuse leads to temperature increasing, self-heating, chain reactions, cumulation of energy and gas, and explosions. An important aspect is the so-called combustion triangle, which determines the coexistence of oxygen, energy, and fuel in the battery system. Too much oxygen in the system (e.g., from components) and the generated heat result in thermal escape. To overcome these problems, appropriate management or countermeasures are implemented. Currently, the five main safety challenges of LIBs are ignition and propagation, standards and regulations, detection and reliability, emergency response, and transport and end-of-life. This work focused on changes in the electrolyte used in the Li-ion cell. A review of non-flammable electrolytes was presented, as well as conventional systems with the addition, usually 5–15%, of a flame retardant, which prevents self-ignition and increases the ignition temperature. In the literature to date, there have been no reviews of the flammability testing methods used and of all types of flammable and non-flammable electrolytes.

Non-flammable electrolytes are certainly closely related to ionic liquids, whose addition results in a huge improvement in safely; liquids containing the bis(trifluoromethylsulfonyl)imide anion have turned out to be especially popular in testing. It comes in a configuration with many cations, acting very positively for the reduction of the spontaneous combustion limit, while obtaining acceptable values of ionic conductivity or charge/discharge capacity. Other ionic liquids are those with a bis(oxalate)borate cation and tetrafluoroborate, which also perfectly fulfill their role in the electrolyte. LiBOB is used both as an electrolyte base and in small amounts as an addition. However, it should be remembered that non-flammable electrolytes are not only ionic liquids, but also systems containing sulfolane, organic phosphates or phosphonates, and polyphosphonates. This shows that the issue of electrolyte safety is a wide field for action, and future research will continue to create new, more secure subsequent layouts. Further works on lithium-ion batteries cells are needed as the electronics market and the miniaturization of devices create a need for efficient, effective, and, above all, safe batteries, which is certainly influenced by their electrolytes.

## Figures and Tables

**Table 1:** Properties of the most commonly used non-aqueous conductive salts in LIBs.

Salt	T_decomposition_ in the Solvent/°C	Al-Corrosion	Conductivity (1 M, EC/DMC, 25 °C)/mS cm^−1^	Electrochemical Stability	Characteristics
LiClO_4_	>100	No	8.4	4.5 V vs. Li/Li+	Insensitive to hydrolysis; no HF is formed; explosive; favorable SEI-forming properties; high thermal/electrochemical stability
LiAsF_6_	>100	No; Al passivates (current collector)	11.1	4.5 V (cathode)/6.3 V (anode) vs. Li/Li^+^	Good SEI formation; toxic degradation products; improves the efficiency of Li metal plating/stripping
LiBF_4_	>100	No	4.9		Strong Lewis base; breaks down and forms HF; less susceptible to hydrolysis and more thermally stable than LiPF_6_
LiPF_6_	>70	Inhibits corrosion of Al	10.7	4.8 V vs. Li/Li^+^	Very sensitive to hydrolysis; stable SEI formation with graphite electrodes; low thermal stability
LiN(SO_2_F)_2_	>100	Yes; small corrosion of Al	>10	4.8 V vs. Li/Li^+^	Insensitive to hydrolysis; does not form HF; expensive in production

**Table 2:** A list of new solvents with their improved characteristics as compared to conventional solvent components, where +/− means positive influence on the electrochemical system and negative and o means no effects.

Solvent	EC	PC	DMC	DEC	EMC
Boiling point	+	+	−	−	-
Melting point	+	−	o	−	−
Dielectric constant	+	+	−	−	−
Viscosity	+	+	−	−	−
Construction to SEI	+	−	−	−	−
Anodic stability	+	+	o	o	o
Safety	+	o	−	−	−
Flash point	+	+	−	−	−

**Table 3:** Composite electrolytes and their ionic conductivity for the application in LIBs.

Polymer/Ceramic/Li Salt	Ionic Conductivity/S cm^−1^	References
PEO-SN/LiTFSI + PAN/LATP/LiTFSI	1.31 × 10^−4^	[74]
PVDF/LATP/LiTFSI	3.31 × 10^−4^	[75]
PEO/LATP/LiClO_4_	1.70 × 10^−4^	[76]
VDF-HFP/LAGP/LiTFSI + EMITFSI	9.60 × 10^−4^	[77]
PEO/LAGP/LiTFSI	8.00 × 10^−4^	[78]
PEGDA/LiTFSI + PAN/LAGP/LiTFSI	3.70 × 10^−4^	[79]
PEO/LLTO/LiClO_4_	2.30 × 10^−4^	[80]
EO/LLZTO/LiClO_4_	9.60 × 10^−4^	[81]
PVDF-HFP/LLZTO/LiTFSI	9.50 × 10^−4^	[82]
PEO/LiTFSI + LATP/PAN/LiTFSI	6.26 × 10^−4^	[83]

**Table 6:** Standardized methods for FS examination.

Method	Description
Pensky–Martens (closed-cup)	FPs above 40 °C, standard volume 68–70 mL; the sample is stirred and the ignition is detected in the automated system via thermocouples as a sudden rise in temperature
Abel (closed-cup)	FPs in range of −30 to 70 °C, standard volume 71–84 mL; the sample is stirred and the ignition is detected in the automated system via thermocouples as a sudden rise in temperature
Cleveland (open-cup)	FPs higher than 79 °C, standard volume 70–80 mL; the sample is not stirred and the ignition is detected in the automated system via ionization systems

**Table 7:** Standardized methods for the FS examination.

Method	
Flash Point	
Advantages	Limitations
Broader range of application, reduced chance of injury, reduced cost of protection, simplified solvent storage. Closed cup—easy to automate, accurate flash points Open cup—quick test, high precision, low price, widely used	Closed cup—leakage of volatile components during flame approach, large temperature difference between vapors and sample, large amount of sample required for the test (50–80 mL). Open cup—higher flash points compared to closed cup (leakage of the vapors)
**SET**	
**Advantages**	**Limitations**
For the SET testing of electrolytes, the solid sample is exchanged with the liquid sample immobilized in a porous carrier material such as glass fiber mats. Many other variants of SET tests with immobilized liquids have been used. As an alternative to the SET tests with immobilized liquids, there are some rare reports on SET tests performed directly on pure liquids. SET tests based on the ignition of pure liquid solvents will provide better reproducibility and comparability than the SET tests with immobilized electrolytes.	No standardized procedure to measure the SETs, which makes it difficult, if not impossible, to compare the SET values obtained from different sources. A specific problem for LIB electrolytes and their components is the lack of complete SET data. SETs are usually published for full electrolyte mixtures and rarely for the components.
**DSC**	
**Advantages**	**Limitations**
Very high processing temperatures, high sensitivity of the instruments, flexibility in crucible volume/form, characteristic transition or reaction temperatures may be accurately determined, stability of the material, simplicity, small sample masses, versatility, short imaging time, more widely available	Uncertainty of heats of fusion, transition, and reaction estimations are in the range of 20–50%, low selectivity for 2-phase mixtures, difficulties with test cell preparation in avoiding volatile solvents, difficult interpretation of data, impossible quantitative analysis and optimization of both high sensitivity and resolution in one experiment, sensitive to changes

**Table 8:** Flammability of different electrolytes used for electrolytes in LIBs in the years 1998–2018.

Electrolyte and FR	Flame Test	Characteristics	References	Year
1 M LIPF_6_ in EC + DMC with 5% of: TFP TTFPi TFMP PFPN FPPN	SET	The five FRs (tris(2,2,2-trifluoroethyl) phosphate (TFP), tris(2,2,2-trifluoroethyl) phosphite (TTFPi), bis(2,2,2 trifluoroethyl) methylphosphonate (TFMP), (ethoxy)pentafluorocyclotriphosphazene (PFPN), and (phenoxy)pentafluoro-cyclotriphosphazene (FPPN)) are investigated as flame retardants. All FR additives remain chemically stable for weeks. Electrochemical system: mesocarbon microbeads-based graphite (anode), Ni_1/3_Co_1/3_Mn_1/3_O_2_ (cathode)	[126]	2018
0.6 M LiBOB in DMMP Ph_3_N DBDB TEDBPDP	Flash point	3,5-di-tert-butyl-1,2-dimethoxybenzene (DBDB), tetraethyl-2,5-di-tertbutyl-1,4-phenylene diphosphate (TEDBPDP) used to ensure thermal safety of electrolytes Electrochemical system: LiMn_2_O_4_ (cathode)	[129]	2016
Modelling	Modelling	The proposed battery pack has high thermal performance for ambient temperatures up to 48 °C for electric vehicles application Electrochemical system: 20 graphite battery pack (anode), LiCoO_2_ (cathode)	[130]	2014
Modelling	Modelling	The efficiency of cooling plates for electric vehicle batteries can be improved by optimizing the geometry of internal fluid channels. Temperature uniformity is most sensitive to the operating conditions	[131]	2014
1 M LiPF_6_ in EC:DEC TPP	Flash point SET	Triphenyl phosphate (TPP) is not considered to be a suitable flame retardant for high power applications Electrochemical system: graphite (anode), LiFePO_4_ (cathode)	[132]	2014
LiBF_4_; LiN(CF_3_SO_2_)_2_ IL	DSC	Good electrochemical stability, ionic liquid (IL) with polymeric matrix increases thermal stability	[133]	2014
1 M LiPF_6_/EC:DMC FR	SET	An efficient phosphaphenanthrene-based (FR) flame retardant, which has low concentrations, high conductivity, and good flame retardant ability	[134]	2014
Modelling	Modelling	Thermal behavior: Volumetric ratio of cooling channel to battery (α) needs to be higher than 0.014 when the inlet Reynolds number of cooling air is around 2000 or higher with a high discharging rate of 2 C	[135]	2013
	Modelling experimental	A two-dimensional CFD (computational fluid dynamics) model has been developed to perform detailed simulations of the thermal management issues within a battery pack cooled by air Electrochemical system: 8 cylindrical commercial cells	[136]	2013
Polymer electrolyte	Modelling calorimetry	According to simulations, the major contributions to the irreversible heat source are enthalpy heating (55–70%) and Joule (30–45%) Electrochemical system: carbon (anode), LiMn_2_O_4_ (cathode)	[137]	2013
Modelling	Modelling	Thermal management analysis: air and silicon oil were selected as cooling media in the battery pack for two conventional flow arrangements, U- and Z-configurations	[138]	2012
1 M LiPF_6_ EC + DEM Pyr_14_TFSI	TGA Flame test	Hydrophobic ionic liquid N-butyl-N-methylpyrrolidinium bis(trifluoromethanesulfonyl)imide (Pyr14TFSI) used with classic liquid electrolyte. Ionic liquid may act as a flame retardant. The lowest amount of Pyr14TFSI needed to observe flame inhibition was 30 wt.%, and with 50 wt.% of IL the tendency to ignite was significantly reduced	[139]	2011
1 M LiPF_6_ EC + DEC TAC, TAIC	SET	Triallyl cyanurate (TAC) and triallyl isocyanurate (TAIC) are new electrolytic additives. TAC is the better thermal protector. Addition of 3 wt.% TAC delays the exothermic reaction by 52 °C, i.e., from 275 to 327 °C. The 5 wt.% TAC solution suppresses 75.1% of exothermic energy from the oxygen evolution reaction Electrochemical system: LiNi_1/3_Mn_1/3_Co_1/3_O_2_ (cathode)	[140]	2011
No data	Modelling	Thermal modeling of a cylindrical LiFePO_4_/graphite lithium-ion battery Electrochemical system: graphite (anode), LiFePO_4_ (cathode)	[141]	
MEE trimer + LiCF_3_SO_3_ MEE trimer + PC/LiCF_3_SO_3_ MEEP + PC/LiCF_3_SO_3_	Flame test Fiber test	Methoxyethoxyethoxyphosphazenes as ionic conductive fire-retardant additives: hexa(methoxyethoxyethoxy)cyclotriphosphazene (MEE trimer), poly(bis(methoxyethoxyethoxy)phosphazene) (MEEP)	[142]	2010
LiBF_4_ + LiBF_2_(C_2_O_4_) PE, EC, DMC (or anhydrous acetonitrile), and IL/poly(ethylene) oxide	DSC	Mixture of LiBF4 and lithium difluoro(oxalato)borate (LiBF_2_(C_2_O_4_)) for application as a new electrolyte. The borate gives a stable SEI layer of low impedance, and it appears that, in a mixture with LiBF_4_, an electrolyte of high conductivity may be achieved	[143]	2010
1 M LiPF_6_in a 1:1 mixture of EC:DMC	Thermodynamic modelling	Control of temperature changes during cell operation (charging/discharging) as a battery control tool Electrochemical system: LTO, Graphite (anode), nanosized LiFePO_4_, LiNi_x_Mn_x_Co_x_O_2_, Li_1.156_Mn_1.844_O^4^ (cathode)	[144]	2010
1 M LiPF_6_/EC + DMC	FTIR calorimetry	Thermal stability of commercial LiPF_6_-based electrolyte is investigated by in situ FTIR spectroscopy along with C80 calorimetry. LNM3O has the worst thermal stability, with a much lower onset temperature and more heat generation below 225 °C. L333 has a good thermal characteristic with a reaction heat below 225 °C Electrochemical system: Li (anode), Li_x_CoO_2_, Li_x_ Ni_0.8_Co_0.15_Al_0.05_O_2_, Li_x_Ni_1/3_Co_1/3_Mn_1/3_O_2_, Li_x_Mn_2_O_4_, Li_x_Ni_0.5_Mn_0.5_O_2_, Li_x_Ni_0.5_Mn_1.5_O_4_, and Li_x_FePO_4_ (cathode)	[145]	2009
1 M LiPF_6_ in organic solvents	Thermodynamic modelling	Overpotential resistance, entropy change, battery heat capacity, and heat transfer coefficient to the ambient air from a battery attached with charge/discharge lead wires were obtained, which are needed to describe the battery thermal behavior Electrochemical system: graphite (anode), LiCoO_2_ (cathode)	[146]	2006
LiTFSI + PEO Middle MW cyclic phosphate	DSC SET	Middle MW cyclic phosphate acts as both the plasticizer and the flame-retarding additive. MW cyclic phosphate gives an opportunity for EV/HEV application	[13]	2006
1.2M LiPF_6_ EC:PC:EMC	Flame test Thermal ramp experiment ARC	Improved technique to evaluate onset temperature, runaway temperature, and the flammability of vent gas and expelled electrolyte in 18,650-size high-power LIBs Electrochemical system: C (anode), LiCo_0.15_Ni_0.8_Al_0.05_O_2_ (cathode)	[147]	2005
LiTFSI in EMC + EC	DSC/TGA	Novel phosphorus-based electrolytes—inherently practical, safe, and non-flammable	[148]	2004
1 M LiPF_6_ EC:PC:EMC TFP	Flammability test	TFP was not flammable by itself; when added to the electrolyte, it reduced its flammability substantially	[149]	2002
LiPF_6_/PAN/EC/PC	Burning test TGA Flame test	The gel electrolyte shows a remarkable fire-retardance	[150]	1998

**Table 9:** Flammability and electrochemical performance of different electrolytes systems and different additives used for electrolytes in LIBs in the years 2001–2020.

Anode	Cathode	Electrolyte	Flame Test	Electrochemical Performance	Coulombic Efficiency	References	Year
Graphite	LiFePO_4_	IE (LiAlC_4_ with xSO_2_, where x = 1 to 22 moles)	Flash point	1.08 Ah shows ultrahigh longevity (50,000 cycles at 2 C up to 20% residual capacity)	99.99%	[151]	2020
	LiFePO_4_	1 M LiPF_6_ in EC: EMC DPMB)	SET	The half-cells containing 1-diphenylphosphoryloxy4-methylbenzene (DPMB)-1, DPMB-2, and DPMB-3 at 1 C achieved values of discharge capacities of ~150 mAh g^−1^ after 100 cycles.	As much as 98%	[12]	2020
Li (lithium)	LiFePO_4_	LiN(SO_2_CF_3_)_2_ (LiTFSI) Intrinsic silicon/phosphorus co-flame retardant polymer solid electrolyte	SET	The cell exhibited a specific capacity of 129.2 mAh g^−1^ at 0.2 C after 100 cycles	70%	[152]	2020
Graphite	LiNi_x_MnyCo-_1-x-y_O_2_ (NMC)	1 M LiPF_6_ in EC + EMC	SET	140 mAh g^−1^ (after 300 cycles at C/20)		[153]	2019
		10% TEPa		125 mAh g^−1^ (after 50 cycles at C/20)	99.5%		
		10% TEPa + 2% VC		110 mAh g^−1^ (after 50 cycles at C/20)	99.6%		
		1.2 M LiFSI in TEPa-BTFE		100 mAh g^−1^ (after 50 cycles at C/20)	99.3%		
		1.2 M LiFSI in TEPa-EC-BTFE		150 mAh g^−1^ (after 300 cycles at C/20)	100%		
LiNiO_2_		Tr1 M LiPF_6_ in TMS + 10% VC	Flash point	195 and 140 mAh g^−1^ (after 20 cycles at C/10)	95%	[154]	2018
LiFePO_4_		LiAlCl_4_ 3SO_2_ (IE)	Flammability test	∼80 mAh g^−^^1^ (after 25 cycles at 10 C)	93.7%	[155]	2018
Graphite	LiCo_1/3_Mn_1/3_ Ni_1/3_O_2_	LiBOB GBL F-EPE	Flash point and flammability test	107.9 mAh g^−1^ (after 500 cycles at 1 C)	80.60%	[156]	2017
Graphite	Li(Ni_0.5_Co_0.2_Mn_0.3_)O_2_	1 M LiPF_6_ in FEC/FEMC (1:9 vol.)	Viscosity test	Comparison of the low efficiency of the EC/EMC system (1/9) with the FEC/FEMC mixture (1/9), which also confirms the instability for the EC/EMC system (1/9) at 4.7 V.	Higher than 81%	[157]	2017
Graphite	LiCoO_2_ (LCO)	1 M LiPF_6_ in EC/DFSM2/EMC (2/3/5 vol.) + 5 wt.% FEC	DSC	Graphite attains a reversible capacity of around 340 mAh g^−1^ (after 50 cycles at 0.1 C). Full cell: 150 mAh g^−1^ (after 135 cycles).	Half-cell: 92.5% Full cell: 99.8%	[158]	2016
SiO	LiFePO_4_	0.8 M LiPF_6_ in DMMP FEC (10%)	SET	Electrolyte additives for lithium-ion battery electrodes: progress and perspectives		[159]	2015
Li_4_Ti_5_O_12_	LiMn_2_O_4_:Li (Ni_1/3_Co_1/3_ Mn_1/3_)O_2_ (8:2)	1 M LiPF_6_ in EC + EMC TBBA	Valve-flame test	525 mAh at 1 C/1 C	98%	[160]	2014
	Li_0.36_Ni_0.8_Co_0.15_ Al_0.015_O_2_	1.2 M LiFP_6_ EMC FEC	DSC	At a slow rate of C/10 cell showed a discharge capacity of 170 mAh g^−1^. Fluoroethylene carbonate (FEC) was used as co-solvent.	No data	[161]	2014
	Li/Li[Li_0.2_ Mn_0.54_Ni_0.13_ Co_0.13_]O_2_	1 M LiPF_6_ EC/DMC/EMC Pp13TFSI	TGA Flash point Flame test	Above 230 mAh g^−1^ at 20 mA g^−1^ after 60 cycles using N-methyl-N-propylpiperidinium bis(trifluoromethanesulfonyl)imide (Pp13TFSI) as nonflammable electrolyte	80.2%	[162]	2013
Graphite	0.4 Li_2_Mn_0.8_Ni_0.1_ Mo_0.1_O_3_ + 0.6LiNi_0.4_Co_0.2_Mn_0.4_O_2_	1.3 M LiPF_6_ in EC/FEC/EMC/DEC (1:2:2:5 v/v) TPP, EDP, TEP		Initial discharge capacity of 5.5 mAh, after 300 cycles at 0.22 mA cm^−2^ achieved the values: 1 mAh, 2.4 mAh, 2.4 mAh, and 3.1 mAh for no-additive electrolyte, TPP, ethyl diphenylphosphinite (EDP), and triethyl phosphite (TEP), respectively.	42% (TPP) 45% (EDP) 56% (TEP)	[163]	2013
Graphite		LiPF_6_ EC/EMC/DMC DADEPA	DSC	N,N-diallyic-diethyoxyl phosphamide (DAPEDA) used as a flame retardant at 75 mA g^−1^ for graphite half-cell achieved a specific capacity of 330 mAh g^−1^ after 100 cycles.	78.5%	[164]	2013
	LiFePO_4_/Li_4_Ti_5_O_12_	1 M LiPF_6_ in EC + DMC [Py_14_]PF_6_	TGA/DSC	The electrolyte solution retained specific charge capacity over 164 mAh g^−1^ at C/3 after 10 cycles adding N-n-butyl-N-methylpyrrolidinium hexafluorophosphate ([Py_14_]PF_6_).	96%	[165]	2013
	LiFePO_4_/Li_4_Ti_5_O_12_	1 M LiPF_6_ in EC:DMC or in EC:DMC:DEC [Py_14_]PF_6_	Flame test	Specific capacities of 170 mAh g^−1^ for LiFePO_4_ and 175 mAh g^−1^ for Li_4_Ti_5_O_12_ at 35 mA g^−1^	83%	[166]	2013
Li foil	LiNi_0.5_Mn_1.5_O_4_	LiPF_6_ in EC/DEC EMP-TFSI	DSC	1-Ethyl-1-methyl piperidinium bis(trifluoromethanesulfonyl)imide (EMP-TFSI) as a co-solvent allowed obtaining a discharge capacity of 110 mAh g^−1^ at 0.5 C after 50 cycles.	100%	[167]	2013
Graphite	LiFePO_4_	1 M LiPF_6_/EC + DMC (1:1)BMEMAP	DSC	Bis(2-methoxyethoxy)methylallylphosphonate (BMEMAP) was used as a flame retardant additive, specific discharge capacity of 140 mAh g^−1^ at 75 mA g^−1^ after 50 cycles	96% (after 2 cycles)	[168]	2013
	LiNi_0.5_Mn_1.5_O_4_	1.2 M LiPF_6_ EC+EMC [AVIm][TFSI]	TGA	The addition of 3 wt.% 1-allyl-3-vinyl imidazolium bis(trifluoromethanesulfonyl)imide ([AVIm][TFSI]) resulted in high discharge capacity of above 180 mAh g^−1^ after 10 cycles at 0.1 C.	100%	[169]	2013
Li	LiFePO_4_	AlMImTFSI + PC LiTFSI	TGA	The cell showed interfacial stability and stable discharge capacities (151 mAh g^−1^) after 100 cycles at 0.1 C rate in 1-allyl-3-methylimidazolium bis(trifluoromethanesulfonyl)imide (AMIMTFSI) in PC (50 wt.%)–1 M LiTFSI electrolyte.	97.4%	[170]	2013
C	LiMn_2_O_4_	1 M LiPF_6_/EC-EMC RDP	Flame test	Resorcinol bis(diphenyl phosphate) (RDP) was used as flame retardant. The charge/discharge capacity of the cell 9.5 mAh/7.8 mAh at 50 mA g^−1^.	72% (10% of RDP)	[171]	2013
	LiFePO_4_ LiMn_2_O_4_ MCMB	1M LiPF_6_ DMC:EC:EMC PNP	Flame test	A phosphazenic compound triethoxyphosphazen-N-phosphoryldiethylester (PNP) was used as flame retardant. The reversible capacity of MCMB electrode at 50th cycle and 100th cycle could still reach 326 and 300 mAh g^−1^ at 40 mA g^−1^.	99% and 91%, respectively	[172]	2013
Li foil	PAN/S composite	LiPF_6_/EC + EMC DMMP	SET	Dimethyl methylphosphonate (DMMP) was used as a flame retardant in a lithium–sulfur cell. High first discharge capacities from 850 to 910 mAh g^−1^ at 0.1 C after 40 cycles.	73% (at 3C)	[173]	2013
Graphite		1M LiPF_6_ DMC:EC:EMC DMMP, DEEP	SET	Two phosphonate esters: DMMP and diethyl ethylphosphonate (DEEP) were used as flame retardants. Specific capacity of 321 mA h g^−1^ at the 50th cycle in DEEP, 50%. In the DMMP electrolyte, the 1st discharge capacity was very large (1303 mA h g^−1^) and the charge capacity was only 108 mA h g^−1^. The current density was equal to 50 mA g^−1^	100% (DEEP)	[174]	2013
C		1 M LiPF_6_-EC/DMC/Fluorinated Comp. A, B, C, D, E	DSC	2,2,3,3,3-pentafluoropropyl methanesulfonate (A), 4-[(2,2,3,3-tetrafluoropropoxy)methyl]-[1,3]-dioxolan-2-one (B), 4-[2,3,3,3-tetrafluoro-2-(trifluoromthyl)propyl]-[1,3]-dioxolan-2-one (C), Bis(2,2,3,3- tetrafluoropropyl)carbonate (D), 2-[2,3,3,3-tetrafluoro-2-(trifluoromethyl)propyl]-1-oxirane, and (E) were used as flame retardants. After 10th cycle, the discharge/charge specific capacity for A, B, C, E was equal to 340/339, 335/333, 315/314, and 341/337 mAh g^−1^ at 60 mA g^−1^, respectively.	99.6% (A), 99.4% (B), 99.5% (C), 98.8% (E)	[175]	2013
Li	LiFePO_4_	BMIMBF_4/γ_-BL (40/60)-1 M LiBF_4_	DSC	1-butyl-3-methyl-imidazolium tetrafluoroborate (BMIMBF4) was used as ionic liquid and combined with γ-butyrolactone (γ-BL). The cell had a 140.3 mAh g^−1^ discharge capacity without any fading during 20 cycles at 0.1 C.	76% (without VC)	[176]	2012
MCMB	LiFePO_4_	0.9 M LiPF_6_/EC/DMC DEMEMPA	Flame test	A new phosphonamidate, bis(N,N-diethyl)(2-methoxyethoxy)methylphosphonamidate (DEMEMPA) was used as a flame-retardant. The cycling behavior of the cathode in and electrolyte containing various contents of DEMEMPA at a current density of 75 mA g^−1^ was up to 145 mAh g^−1^ after 50 cycles. MCMB anode delivered a capacity of 320 mAh g^−1^ after 10 cycles at 50 mA g^−1^	98.4% (after 5th cycle for anode)	[177]	2012
MCMB	LiFePO_4_	0.7 M LiBOB-SL/DMS 0.7 M LiBOB-SL/DES	EIS, charge–discharge test	Sulfolane (SL), dimethyl sulfite (DMS), and diethyl sulfite (DES) were used as mixed solvents. MCMB: charge and discharge capacities were equal to 253 mAh g^−1^ and 297 mAh g^−1^, respectively, after the first cycle. Cathode: 100 mAh g^−1^ after 100 cycles at 0.5 C. The capacity retention efficiency of the cell with LiBOB-SL/DMS and LiBOB-SL/DES electrolyte was found to be 94.0% and 66.5%, respectively.	85% (anode, 25 °C) 94% (DMS, 60 °C), 66.5% (DES, 60 °C)	[178]	2012
Li_1_ + xMn_2_O_4_	Li_1−x_Mn_2_O^4^	1 M LiPF_6_ in EC:DEC Solid electrolyte Li_1.3_Ti_1.7_Al_0.3_(PO_4_)_3_	Heat test	After 5 cycles, the capacity of the hybrid electrolyte cell dropped quickly and was saturated at ∼30 mAh g^−1^ at 0.1 mA cm^−2^	No data	[179]	2012
Graphite		1 M LiClO_4_ EC/DEC/PC Fluorocarbonates: 10–33 vol%	DSC	After 10 cycles, the half-cell achieved the specific discharge/charge capacity of 313–335/309–331 mAh g^−1^ at 60 mA g^−1^.	98.7–99.2%	[180]	2011
Graphite		1 M LiPF_6_/PC + DMC (1:1) MPBMDS		The addition of 4% methyl phenyl bis-methoxydiethoxysilane (MPBMDS) had little effect on cycling behavior, with electrodes exhibiting reversible capacities above 280 mAh g^−1^ without fading after 30 cycles at 90 mA g^−1^.	70%	[181]	2011
Carbon coating-hard carbon and graphite composite materials	LiFePO_4_ and Li/Li_4_Ti_5_O_12_	MPPyrTFSI EMImTFSI LiTFSI	TGA flame test	The cells using 0.2 M LiTFSI/EMITFSI-PYR13TFSI (1:1, v/v) electrolyte showed reversible capacities of 132 mAh g^−1^ for LiFePO_4_ at 1/15 C, 134 mAh g^−1^ for Li/Li_4_Ti_5_O_12_ at 1/10 C, and 275 mAh g^−1^ at 1/20 C for carbon material after 30 cycles.	90% (Li/Li_4_Ti_5_O_12_), 99% (Li/Li_4_Ti_5_O_12_), 98% (carbon)	[182]	2011
C/Li	LiCoO_2_/Li	1 M LiPF_6_/EC/DEC CDP	Micro-calorimeter	Cresyl diphenyl phosphate (CDP) was used as a flame retardant. The specific capacities for the Li/C cells with 5%, 10%, and 15% CDP content electrolyte were 314.5 mAh g^−1^, 326.5 mAh g^−1^, and 321.1 mAh g^−1^ with standard deviation of 16.2, 10.8, and 8.1 for the 60th, 66th, and 57th cycles, respectively, at 0.2 mA cm^−2^. The LiCoO_2_/Li cell with 20%-CDP electrolyte showed the lowest specific capacity of 124 mAh g^−1^.	No data	[183]	2011
C	LiCoO_2_	1.4 M LiPF_6_ EC + FEC + EMC BMP-PF_6_	SET	1-butyl-1-methylpyrrolidinium hexafluorophosphate (BMP-PF_6_) was used as a flame-retarding additive. First discharge/charge capacity of the cell with 10% of BMP-PF6 was equal to 146.6/156.6 mAh g^−1^ at 0.5 C.	93.6%	[184]	2011
Hard carbon–graphite composite	LiFePO_4_	1 M LiPF_6_/EC-DEC LiBOB, PP_13_TFSI	DSC Flame test	Lithium bis(ÿxalate)borate (LiBOB) was used to enhance stable SEI formation. The anode half-cell showed 200 mA h g^−1^ reversible capacity at 0.3 C and 140 mAh g^−1^ at 0.1 C for the cathode.	68% (anode), 99% (cathode)	[185]	2011
Graphite	LiNi_0.8_Co_0.1_ Al_0.05_O_2_	1M LiPF_6_ in EC:DMC alkylsilane compounds as electrolyte solvents	Flame test	The cell was charged and discharged at a C/5 rate and cycled between 3.0 and 4.2 V at room temperature. It exhibited excellent cycling performance with only 9% capacity loss over 200 cycles (1.35 mAh).	91%	[186]	2010
Graphite	LiCoO_2_	1 M LiPF_6_ in EC + DEC + TPTP 1 M Li-BETI in EC + DEC + TPTP 1 M Li-BETI in DEC + TMMP	Cyclic Voltammetry	Hydrofluoroethers (HFEs) of 2-trifluoromethyl-3-methoxyperfluoropentane (TMMP) and 2-(trifluoro-2-fluoro-3-difluoropropoxy)-3-difluoro-4-fluoro-5-trifluoropentane (TPTP) were examined as cosolvents of ethylene carbonate (EC) +diethyl carbonate (DEC). The obtained discharge capacity at 0.2 C for EC + DEC EC + DEC and EC + DEC + TPTP were 133, 140, and 129 mAh g^−1^ per LiCoO_2_, respectively.	Almost 100% (cathode) 60% (EC + DEC + TMMP, full cell)	[187]	2010
Mesocarbon microbead	LiNi_0.8_Co_0.2_O_2_	1 M LiPF_6_ + 5% LiBOB in EC/EMC/DMMP 1 M 95% LiPF_6_ + 5% LiBOB in EC/EMC/DMMP	SET NMR	15% DMMP with 5% of LiBOB allowed obtaining a discharge specific capacity of 150 mAh g^−1^ after 35 cycles at C/5.	No data	[188]	2010
Graphite	LiFePO_4_/Li4Ti_5_O_12_	1 M LiPF_6_ EC + DEC + IL	TGA Flame test	1-ethyl-3-methylimidazolium-bis(fluorsulfonyl)imide (EMIm-TFSI), propyl-methyl-imidazolium-bis(fluorsulfonyl)imide (PMIm-TFSI), and hexyl-methyl-imidazolium-bis(fluorsulfonyl)imide (HMIm-TFSI) were used as ILs. The first cycle of anode and cathode cycling exhibited discharge/charge specific capacity of maximum 388/337 mAh g^−1^ (20% of IL) at C/24, 154/154 mAh g^−1^ (10% of IL) at C/12, respectively.	87% (anode), 100% (cathode)	[189]	2010
Graphite	LiMn_2_O_4_	0.4 M LiTFSI/PP_13_TFSI EC + TEP	Flame test	For the graphite half-cell, the addition of TEP and EC improved the discharge capacity from 37.5 mAh g^−1^ to 154.2 mAh g^−1^ at 1 C discharge rate and 66.1 mAh g^−1^ to 204.1 mAh g^−1^ at C/5 rate. For the cathode, a high discharge capacity of 99.1 mAh g^−1^ and 64.2 mAh g−1 at high discharge rates at 1 C and 3 C, respectively, were achieved.	No data	[190]	2010
Graphite	LiMn_2_O_4_	1 M LiBF_4_/EC + DEC + TEP PVdF-HFP (host polymer)	TSC	Thermal safety calorimetry (TSC) was used to examine the thermal stability. LiMn_2_O_4_ electrode measured at 0.3 C achieved a specific discharge capacity of 104.5 mAh g^−1^ after the first cycle. The discharge capacity for graphite was equal to 126 mAh g^−1^ for the first discharge, which was lower than the ideal value for this material.	No data	[191]	2009
Li_4_Ti_5_O_12_	LiMn_2_O_4_ LiNi_0.5_ Mn_1.5_O_4_	1 M LiPF_6_/LiTESI TMS + EMC 1:1	Flame test	In sight of imide salt (LiTFSI) and ethyl methyl or tetramethyl sulfone (TMS) electrolytes, the Li_4_Ti_5_O_12_/LiMn_2_O_4_ (1) cell exhibited a specific capacity of 80 mAh g−1 after 100 cycles at C/3. With LiNi_0.5_Mn_1.5_O_4_ (2) and 1 M LiPF_6_ in TMS as electrolyte, the capacity was equal to 110 mAh g^−1^ at C/12. The Li_4_Ti_5_O_12_/LiNi_0.5_Mn_1.5_O_4_ (3) cell achieved an initial capacity of 240 mAh g^−1^ after 1000 cycles under 2 C	99% (1), 99% (2)	[192]	2009
Li	LiCoO_2_	1 M LiPF_6_ in EC/EMC/DMC 1:1:1 VTMS	DSC	Vinyl-tris-(methoxydiethoxy)silane (VTMS) (5–15%) was used as a flame retardant for the electrolyte. Cycling behavior at 1 C after the 40th cycle was 125 mAh g^−1^ (5% vol. of VTMS).	No data	[193]	2009
	LiCo_1/3_ Ni_1/3Mn1/3_O_2_	1.1M LiPF_6_ in EC/EMC (4:6) HMTP HETP	DSC	Hexamethoxycyclo-tri-phosphazene (HMTP) and hexaethoxy-cyclotri-phosphazene (HETP) were added to electrolyte as flame retardants. The HMTP-based electrolyte in the half-cell delivered a discharge capacity of 138 mAh g^−1^ at 0.5 C after 50 cycles (5% of HMTP), while the HETP-based one delivered a capacity of 135 mAh g^−1^ at 0.5 C after 50 cycles (1% of HETP).	No data	[194]	2009
Graphite	LiCoO_2_	1 M LiTFSI + CN[CH_2_]3CN + PC EC (co-solvent) 0.1 M LiBOB (co-salt)	DSC	Glutaronitrile, CN[CH_2_]_3_CN, was evaluated as a co-solvent that is thermally and (anodically) electrochemically stable. The battery showed an initial discharge capacity of 98 mAh g^−1^ that decreased gradually on cycling, reaching a stable value at the 80th cycle and beyond up to the 100th cycle at C/12, with	74%	[195]	2009
Graphite	LiNi_0.3_Mn_0.3_Co_0.3_O_2_	1 M LiPF_6_ EC:DEC TPP	DSC	1.3 mAh at C/2 rate with 3% of the TPP-additive after the 40th cycle.	89%	[196]	2007
MCMB	LiCoO_2_	1.1M LiPF_6_ EC/EMC + TPP	DSC	Blank electrolyte +3% TPP-based cell showed a discharge/charge specific capacity of 125.7/129 mAh g^−1^ at 0.5 C.	97.4%	[197]	2007
MCMB/surface-modified graphite (SMG)	LiCoO_2_	1 M LiPF_6_/EC + DEC + DMMP	SET	Dimethyl methylphosphonate (DMMP) was used as a co-solvent. The cathode delivered a specific discharge capacity of 132 mAh g^−1^ after 30 cycles at 0.2 mA cm^−2^. The anodes exhibited the discharge capacity of 170 mAh g^−1^ and 200 mAh g^−1^ at 0.2 mA cm^−2^ after the 1st cycle for MCMB and SMG, respectively.	85.8% (MCMB), 88.2% (SMG)	[198]	2007
SMG	LiCoO^2^	1 M LiPF^6^/EC + DEC DMMP	SET	LiCoO^2^/Li and LiCoO^2^/graphite cells were cycled at 0.20 mA cm^−2^ initially and 0.65 mA cm^−2^ after the 3rd cycle. The specific capacities were equal to 125 mAh g^−1^ after 30 cycles (10% of DMMP) and 3.5 mAh after 20 cycles (10% of DMMP) for the half-cell and the full-cell, respectively.	No data	[199]	2007
	LiCo_0.8_ Co_0.2_O_2_	1 M LiPF_6_ EC + DEC + TMP	Flammability test	A comparative study was performed for trimethyl phosphite (TMP(i)) and trimethyl phosphate (TMP(a)) as electrolyte additives. The cell was cycled at the current density of 0.1 mA cm^−2^ in the first three cycles, and then at 0.2 mA cm^−2^. The discharge specific capacity was equal to 130 mAh g^−1^ after 35 cycles (5% of TMP(a) addition) and for TMP(i) approx. 150 mAh g^−1^ after 42 cycles.	No data	[200]	2005
Graphite	LiCoO_2_	DEME-TFSI + LiTFSI	TGA	A novel aliphatic quaternary ammonium type ionic liquid, N, N-diethyl- N-methyl- N-(2-methoxyethyl)ammonium bis(trifluoromethylsulfonyl)imide (DEME-TFSI), was used as an electrolyte. The graphite/Li–DEME–TFSI containing 10 wt.% of VC/LiCoO_2_ cell showed a specific capacity of 122 mAh g^−1^ after 100 cycles at 0.1 C.	Over 99%	[167]	2004
Graphite	LiNi_0.8_Co_0.2_O_2_	1 M LiPF_6_ EC:DEC + FR TPP TBP	Flame test	Triphenylphosphate (TPP) and tributylphosphate (TBP) were used as flame-retardants (FRs) to provide superior thermal safety in lithium-ion cells at the fully charged state. The specific discharge capacity of the cell with 5% of TPP and 5% of TBP was equal to 120, 100 mAh g^−1^ after 150 cycles, respectively, at C/20.	No data	[201]	2003
Graphitic composite	LiNiO^x^ (HEV)	1 M LiPF^6^ in EC + EMC + FR TFP BMP TDP TMP TEP HMPN	SET	Tris(2,2,2-trifluoroethyl) phosphate (TFP), bis(2,2,2-trifluoroethyl)methyl phosphate (BMP), (2,2,2-trifluoroethyl)diethyl phosphate (TDP), hexamethyl phosphazene (HMPN), phosphates trimethyl phosphate (TMP), and triethyl phosphate (TEP) were used as FRs. The specific capacity of the cell with 40% of TFP was equal to 2.3 mAh cm^−2^ after the 10th cycle at 0.35 mA cm^−2^.	99%	[202]	2003
Graphite	LiNiO_x_ (HEV)	1 M LiPF_6_ in EC/EMC + FR 1 M LiPF_6_ in PC/EC/EMC + FR TFP BMP	Galvanostatic test	The discharge specific capacity of the cell with TFP and BMP (15%) was equal to 1.9 mAh cm^−2^ after 250 cycles and 1.9 mAh cm^−2^ after 160 cycles at 2.91 mA cm^−2^, respectively.	98.9% (40% BMP after the 2nd cycle), 100% (40% of TFP after the 2nd cycle)	[203]	2003
MCMB Li	LiCo O_2_, LiNi_0:8_Co_0:2_O_2_	1 M LiPF_6_ in EC/DMC LiV_6_O_13_/LiTFSI in oxymethylene-linked poly(ethylene oxide) (PEMO)	Heat of mixing (thermodynamic modelling)	The heat was measured for the “liquid” cell (with 1 M LiPF_6_) previously cycled at 12.29 A m^−2^ and “polymeric” cell at 10 A m^−2^. The entropy of reaction accounts for a reversible heat effect, which may be of the same order of magnitude as the resistive heating.	No data	[204]	2003
Synthetic graphite	LiCoO_2_	1 M LiBETI/1 M LiPF_6_ EC:PC:BC:EMC MFE	Flash point PO_4_- generation	Nonflammable methyl nonafluorobutyl ether (MFE) was used as an electrolyte. 1 M LiBETI-MFE/EMC in LiCoO_2_/Li cell showed the charge/discharge capacity of 137/134 mAh g^−1^ at 0.28 mA cm^−2^ after the 1st cycle. In the graphite/Li cell, the values achieved 99/74 mAh g^−1^ at the same cycling conditions.	97%, 75%	[205]	2003
Li (Rayovac BR 2335 button cell)	Li (Rayovac BR 2335 button cell)	1 M LiPF^6^ EC:EMC TMP TEP HMPN	Flammability test	All cells were cycled at the current density of 0.35 mA cm^−2^. The TMP-based cell (10%) achieved a specific capacity of 0.65 mAh cm^−2^ after approx. 80 cycles while the cell with 10% of TEP achieved 0.8 mAh cm^−2^ after approx. 120 cycles and with 5% of HMPN achieved 1.15 mAh cm^−2^ after 60 cycles.	10%, 65%, 100% (after 1st cycle), respectively	[206]	2002
Graphite	LiNiO_x_	1 M LiPF_6_ EC + EMC + FR TFP	SET	The cell with 50% of TFP was cycled at 0.36 mAh cm^−2^ achieving a specific capacity of 2.3 mAh cm^−2^ after the 10th cycle.	96%	[207]	2002
Graphite	LiCoO_2_	LiBETI LiTFSI MFE	Flash point	The cell assembled with 1 M LiBETI–MFE/EMC discharged the designed capacity (1400 mAh) at 0.1 C after 50 cycles	80%	[208]	2002
MCMB	Li_0.5_CoO_2_	LiPF_6_ in EC:DEC	DSC ARC XRD	assuming a first discharge capacity of approx. 350 mAh g^−1^ for the anode and approx. 140 mAh g^−1^ for the cathode at 4.2 V.	No data	[209]	2001
Natural graphite	LiCoO_2_	1 M LiPF_6_ in EC + PC + DEC TMP	Flame test	After the 1st one we may observe that the TMP content should be limited to <10% for the EC:PC:TMP electrolyte or <25% for the EC:DEC:TMP electrolyte. For EC:PC:TMP the TMP stability was achieved for 45:45:10 with a discharge capacity of 268 mAh g^−1^ and for EC:DEC:TMP for 40:40:20 with a capacity of 245 mAh g^−1^ at 0.2 mA cm^−2^.	84%, 81%	[210]	2001
Amorphous carbon (AC) Natural graphite	LiCoO_2_	1 M LiPF_6_ EC + PC + DEC TMP		The AC/LiCoO_2_ ion cell with 20% of TMP at a charge/discharge current density of 0.1/0.2 mA cm^−2^ achieved a specific charge/discharge capacity of 150 mAh g^−1^/114 mAh g^−1^ at the first cycle.	76%	[211]	2001

**Table 10:** Advantages and disadvantages of hydrogen propulsion in bus transport [221].

ADVANTAGES
Ecological
Low noise level
Short charging time and high range
High efficiency despite high price
Economic benefits
DISADVANTAGES
High production costs
High infrastructure costs
The need to invest in training and communication

**Table 11:** Advantages and disadvantages of hydrogen propulsion in railway transport [221].

ADVANTAGES
Ecological
Economic benefits
Flexibility and versatility
High efficiency despite high price
Short charging time and high range
DISADVANTAGES
Fuel costs
Logistics and distribution costs
Technical difficulties with the vehicle construction
Relatively low service life of fuel cells

## Data Availability

The data are available on request.

## Abbreviations

ΔH	Enthalpy
Q	Heat flow
T	Temperature
EC	Ethyl carbonate
DMC	Dimethyl carbonate
Al	Aluminum
LiClO_4_	Lithium perchlorate
LiAsF_6_	Lithium hexafluoroarsenate (V)
LiBF4	Lithium tetrafluoroborate
LiN(SO_2_F)_2_ (LISFI)	Lithium bis(fluorosulfuryl)imide
POF_3_	Phosphoryl fluoride
PF_5_	Phosphorus pentafluoride
LLZTO	Li_6.4_La_3_Zr_1.4_Ta_0.6_O_12_
EO	Ethylene oxide
LLTO	Li_0.33_La_0.557_TiO_3_
PAN	Poly(acrylonitrile)
BisAEA_4_	Ethoxylated bisphenol A diacrylate
LiFePO_4_	Lithium iron phosphate
MPPipTFSI	N-methyl-N-propylpiperidinium bis(trifluoromethanesulfonyl)imide
LiNfO	Lithium nonafluoro-1-butanesulfonate
EMImNfO	1-ethyl-3-methyl imidazolium nonfluoro-1-butanesulfonate
PYR_14_-TFSI	1-butyl-1-methyl pyrrolidinium bis(trifluoromethylsulfonyl)imide
SiO_2_-PAALi	Silicon dioxide–ion complex
LiCoO_2_	Lithium cobalt oxide
CO	Carbon monoxide
CH_4_	Methane
C_2_H_4_	Ethylene
C_2_H_6_	Ethane
C_3_H_6_	Propene
HDV	High duty vehicle
DEC	Diethyl carbonate
CO_2_	Carbon dioxide
Et_2_O	Ethyl ether
SN	Succinonitrile
LATP	NASICON-type lithium aluminum titanium phosphate
LiTFSI	Lithium bis(trifluoromethanesulfonyl)imide
PVDF	Polyvinylidene fluoride
HFP	Hexafluoropropylene
EMITFSI	Lithium bis(trifluoromethanesulfonyl)imide
LAGP	NASICON-type Li_1.5_Al_0.5_Ge_1.5_(PO_4_)_3_
PEGDA	Polyethylene glycol diacrylate
PFPN	Ethoxy(pentafluoro)cyclotriphosphazene
FPPN	Pentafluoro(phenoxy)cyclotriphosphazene
LiCoO_2_	Lithium cobalt oxide
C_3_H_8_	Propane
TMP	Trimethyl phosphate
TEP	Triethyl phosphate
TBP	Tributyl phosphate
TFP	Tris(2,2,2-trifluoroethyl) phosphate
BMP	Bis(2,2,2-trifluoroethyl) methylphosphonate
TMPi	Trimethyl phosphite
TTFPi	Tris(2,2,2-trifluoroethyl) phosphite
DMMP	Dimethyl methylphosphonate
DEEP	Diethyl ethylphosphonate
TFMP	Bis(2,2,2-trifluoroethyl) methylphosphonate
TFEP	Bis(2,2,2-trifluoroethyl) ethylphosphonate
HMOCPN	Hexamethoxycyclotriphosphazene
NMP	N-methyl-2-pyrrolidone
CSR	Corporate social responsibility
